# *De novo* mutations in *GRIN1* cause extensive bilateral polymicrogyria

**DOI:** 10.1093/brain/awx358

**Published:** 2018-01-22

**Authors:** Andrew E Fry, Katherine A Fawcett, Nathanel Zelnik, Hongjie Yuan, Belinda A N Thompson, Lilach Shemer-Meiri, Thomas D Cushion, Hood Mugalaasi, David Sims, Neil Stoodley, Seo-Kyung Chung, Mark I Rees, Chirag V Patel, Louise A Brueton, Valérie Layet, Fabienne Giuliano, Michael P Kerr, Ehud Banne, Vardiella Meiner, Tally Lerman-Sagie, Katherine L Helbig, Laura H Kofman, Kristin M Knight, Wenjuan Chen, Varun Kannan, Chun Hu, Hirofumi Kusumoto, Jin Zhang, Sharon A Swanger, Gil H Shaulsky, Ghayda M Mirzaa, Alison M Muir, Heather C Mefford, William B Dobyns, Amanda B Mackenzie, Jonathan G L Mullins, Johannes R Lemke, Nadia Bahi-Buisson, Stephen F Traynelis, Heledd F Iago, Daniela T Pilz

**Affiliations:** 1 Institute of Medical Genetics, University Hospital of Wales, Cardiff CF14 4XW, UK; 2 Division of Cancer and Genetics, School of Medicine, Cardiff University, Cardiff CF14 4XN, UK; 3 MRC Computational Genomics Analysis and Training Programme (CGAT), MRC Centre for Computational Biology, MRC Weatherall Institute of Molecular Medicine, John Radcliffe Hospital, Headington, Oxford OX3 9DS, UK; 4 Pediatric Neurology Unit, Carmel Medical Center, Haifa, Israel; 5 Bruce and Ruth Rappaport Faculty of Medicine, Technion, Haifa, Israel; 6 Department of Pharmacology and Chemical Biology, Emory University School of Medicine, Atlanta, GA 30322, USA; 7 Center for Functional Evaluation of Rare Variants (CFERV), Emory University School of Medicine, Atlanta, GA 30322, USA; 8 Department of Pharmacy and Pharmacology, University of Bath, Claverton Down, Bath BA2 7AY, UK; 9 Department of Neuroradiology, North Bristol NHS Trust, Frenchay Hospital, Bristol BS16 1LE, UK; 10 Neurology and Molecular Neuroscience Research, Institute of Life Science, Swansea University Medical School, Swansea University, Swansea SA2 8PP, UK; 11 Genetic Health Queensland, Royal Brisbane and Women’s Hospital Campus, Herston, Brisbane, Queensland 4029, Australia; 12 West Midlands Regional Genetics Service, Clinical Genetics Unit, Birmingham Women’s Hospital, Birmingham B15 2TG, UK; 13 Service de Génétique Médicale, Groupe Hospitalier du Havre, Hôpital Jacques Monod, Le Havre, France; 14 Service de Génétique Médicale, Centre Hospitalier Universitaire de Nice, Nice, France; 15 MRC Centre for Neuropsychiatric Genetics and Genomics, Institute of Psychological Medicine and Clinical Neurosciences, Cardiff University, Cardiff CF24 4HQ, UK; 16 Learning Disabilities Directorate, Abertawe Bro Morgannwg University NHS Trust, Treseder Way, Caerau, Cardiff CF5 5WF, UK; 17 Clinical Genetics Institute, Kaplan Medical Centre, Rehovot, Israel; 18 Department of Genetics and Metabolic Diseases, Hadassah-Hebrew University Hospital, Jerusalem, Israel; 19 Pediatric Neurology Unit, Wolfson Medical Centre, Holon, Sackler School of Medicine, Tel-Aviv University, Tel-Aviv, Israel; 20 Division of Neurology, Children’s Hospital of Philadelphia, Philadelphia, PA 19104, USA; 21 Kaiser Permanente Mid-Atlantic States, McLean, VA 22102, USA; 22 Department of Neurology, Xiangya Hospital, Central South University, Changsha 410013, China; 23 Department of Neurology, the First Hospital of Shanxi Medical University, Taiyuan, 030001, China; 24 Division of Genetic Medicine, Department of Pediatrics, University of Washington, Seattle, WA 98195, USA; 25 Center for Integrative Brain Research, Seattle Children’s Research Institute, Seattle, WA 98195, USA; 26 Department of Neurology, University of Washington, Seattle, WA 98195, USA; 27 Genome and Structural Bioinformatics Group, Institute of Life Science, Swansea University, Singleton Park, Swansea SA2 8PP, UK; 28 Institute of Human Genetics, University Medical Center Leipzig, Leipzig 04103, Germany; 29 Imagine Institute, INSERM UMR-1163, Laboratory Genetics and Embryology of Congenital Malformations, Paris Descartes University, Paris, France; 30 West of Scotland Clinical Genetics Service, Queen Elizabeth University Hospital, Glasgow G51 4TF, UK

**Keywords:** polymicrogyria, *GRIN1*, GluN1, NR1, *N*-methyl-d-aspartate receptor

## Abstract

Polymicrogyria is a malformation of cortical development. The aetiology of polymicrogyria remains poorly understood. Using whole-exome sequencing we found *de novo* heterozygous missense *GRIN1* mutations in 2 of 57 parent-offspring trios with polymicrogyria. We found nine further *de novo* missense *GRIN1* mutations in additional cortical malformation patients. Shared features in the patients were extensive bilateral polymicrogyria associated with severe developmental delay, postnatal microcephaly, cortical visual impairment and intractable epilepsy. *GRIN1* encodes GluN1, the essential subunit of the *N*-methyl-d-aspartate receptor. The polymicrogyria-associated *GRIN1* mutations tended to cluster in the S2 region (part of the ligand-binding domain of GluN1) or the adjacent M3 helix. These regions are rarely mutated in the normal population or in *GRIN1* patients without polymicrogyria. Using two-electrode and whole-cell voltage-clamp analysis, we showed that the polymicrogyria-associated *GRIN1* mutations significantly alter the *in vitro* activity of the receptor. Three of the mutations increased agonist potency while one reduced proton inhibition of the receptor. These results are striking because previous *GRIN1* mutations have generally caused loss of function, and because *N*-methyl-d-aspartate receptor agonists have been used for many years to generate animal models of polymicrogyria. Overall, our results expand the phenotypic spectrum associated with *GRIN1* mutations and highlight the important role of *N*-methyl-d-aspartate receptor signalling in the pathogenesis of polymicrogyria.

## Teaser

NMDA receptor agonists have been used for many years to generate animal models of polymicrogyria, a malformation of cortical development. Fry *et al.* identify *de novo GRIN1* mutations in eleven patients with severe bilateral polymicrogyria. Polymicrogyria-associated *GRIN1* mutations cluster in specific protein domains and significantly alter NMDA receptor function.

## Introduction

Malformations of cortical development (MCDs) are a spectrum of brain abnormalities that occur due to disruption of the intricate developmental processes that form the cerebral cortex. Although rare, MCDs have a major impact on the lives of patients and their families. Polymicrogyria is a subtype of MCD characterized, macroscopically, by an excessive number of small cortical folds (gyri). At a microscopic level, polymicrogyria is associated with abnormal cortical architecture including thinning or loss of cortical layers (Squier and Jansen, 2014; Jansen *et al.*, 2016). The clinical effects of polymicrogyria depend on the extent and regions of the brain affected. Common consequences include intellectual disability, epilepsy, spasticity and cortical visual impairment (Stutterd and Leventer, 2014). Polymicrogyria can result from non-genetic events such as hypoxic-ischaemic insults or congenital infections. In addition, a range of chromosomal and single-gene disorders have been identified in polymicrogyria patients (Jansen and Andermann, 2005; Stutterd and Leventer, 2014). Despite these discoveries, the underlying cause of the malformation remains unknown in the majority of patients.

Polymicrogyria is usually a sporadic disorder, with most patients having no family history. Polymicrogyria also causes a significant loss of reproductive fitness. This suggests a role for *de novo* mutations in the aetiology of the disorder. Recent candidate gene and exome sequencing studies have shown that *de novo* mutations cause polymicrogyria in some patients (Jaglin *et al.*, 2009; Riviere *et al.*, 2012; Mirzaa *et al.*, 2014). Based on these observations we took the approach of exome sequencing in a cohort of 57 parent-offspring trios. This strategy led us to identify two likely causal variants in *GRIN1* in patients with extensive bilateral polymicrogyria. *GRIN1* encodes GluN1, the obligatory subunit of the *N*-methyl-d-aspartate (NMDA) receptor, an ionotropic glutamate receptor that is highly expressed in the foetal brain (Law *et al.*, 2003). Mutations in *GRIN2B*, a gene that encodes a different NMDA receptor subunit, have recently been reported in MCD patients (Platzer *et al.*, 2017). Therefore, having observed two *de novo GRIN1* mutations in patients with polymicrogyria, we searched for additional *GRIN1* mutations in MCD patients. Furthermore, we examined the functional impact of polymicrogyria-associated *GRIN1* mutations by computer-based protein structure modelling and *in vitro* electrophysiological analysis.

## Materials and methods

### Patients

Patients 1 and 2 were part of a cohort of 57 unrelated proband-parent trios recruited from Clinical Genetic and Paediatric Neurology clinics around the UK. All probands had polymicrogyria demonstrated by MRI and confirmed by review of the neuroradiology. The probands had no known cause for their polymicrogyria and normal array comparative genome hybridization. Parents were judged to be unaffected based on history and a brief physical examination. Most parents had not undergone brain imaging. For more details about the UK cohort see Supplementary Table 1. The study was approved by the Research Ethics Committee for Wales (09/MRE09/51). Informed consent was obtained from all participants (or their parents/legal guardians) prior to testing. Patients 3, 4, and 6–11 were ascertained through a request to collaborators and members of the European Network on Brain malformations looking for similar patients. Patients 3 and 4 underwent trio-based whole exome sequencing as part of an ongoing research program in France. Patients 6–8 had trio-based exome sequencing performed during their clinical diagnostic workup. Patients 9–11 were part of a cohort of 211 polymicrogyria patients who underwent targeted sequencing of the *GRIN1* gene as part of a US-based research program. Patient 5 had trio-based whole exome sequencing performed on a clinical basis. He was ascertained due to appearing in a poster at the European Paediatric Neurology Society Congress 2015.

### Exome sequencing and *in silico* prediction

Standard approaches to exome sequencing and variant filtering were used. Detailed descriptions are given in the Supplementary material. Predictions of the functional impact of the *de novo* mutations were made by a range of analysis programs. These included PhyloP (Pollard *et al.*, 2010), SIFT (Kumar *et al.*, 2009), PolyPhen-2 (Adzhubei *et al.*, 2010), MutationTaster (Schwarz *et al.*, 2014), CADD v1.3 (Kircher *et al.*, 2014) and M-CAP (Jagadeesh *et al.*, 2016). We searched the ExAC database for each variant (release 0.3, 14 January 2016) (Lek *et al.*, 2016). Genomic coordinates are based on genome build hg19/GRCh37 (February 2009). Coding and protein positions of the *GRIN1* mutations are based on GenBank accession codes NM_007327.3 (ENST00000371561.3) and NP_015566.1 respectively.

### Homology modelling

Structural modelling of wild-type and mutant GluN1, and wild-type GluN2A proteins was carried out using a previously-described homology-based modelling pipeline (Mullins, 2012). This approach uses the solved structure of a homologous template to model the native folds of a target sequence. The target sequences selected were wild-type GluN1 (based on Q05586, Uniprot Isoform 3 FASTA file) and wild-type GluN2A (based on Q12879, Uniprot Isoform 1 FASTA file). The template for both Q05568 and Q12879 was the crystal structure of the GluN1a/GluN2B NMDA receptor (4PE5 chain A) from *Rattus norvegicus* (Karakas and Furukawa, 2014). The 4PE5 chain A contains the amino terminal domain, agonist binding domain, and transmembrane domain of the wild-type channel tetramer. We constructed our models using two GluN1 and two GluN2A subunits arranged as 1-2-1-2. Target and template underwent structural and consensus alignment using T-Coffee (Notredame *et al.*, 2000). Homology modelling was performed by MODELLER (Webb and Sali, 2016). The putative structure was refined to improve the accuracy of non-conserved regions, optimize bond geometries and remove unfavourable contacts. Structural models were viewed and analysed using the UCSF Chimera software (https://www.cgl.ucsf.edu/chimera/) (Pettersen *et al.*, 2004). Each mutant receptor was superimposed onto the wild-type model. The effects on the transmembrane helices were assessed by measuring the displacement, between the two models, of residues at the ends of each helix (from alpha carbon atoms). The residues were 559, 581, 615, 606, 655, 630, 810 and 828. The superimposed models were also used to calculate root-mean-square deviation (RMSD) values for nine domains (using all backbone atoms in the specified residues): amino terminal (residues 23–394), first and second ligand binding domains (S1 and S2; residues 395–544 and 658–808, respectively), transmembrane domains one to four (M1–M4; residues 560–580, 606–615, 637–657, and 813–833), DRPEER motif (658–663) and SYTANLAAF motif (646–654). UCSF Chimera FindHBond function (using the default relaxation) was used to predict hydrogen bonding between the glycine ligand and the glycine-binding residues of GluN1. UniProt lists these as 516–518, 523, 688 and 732.

### Expression plasmids and mutagenesis

For two-electrode voltage clamp recordings, the cDNA for human wild-type NMDA subunits GluN1, GluN2A, and GluN2B (GenBank accession codes: NP_015566, NP_000824 and NP_000825, respectively) were subcloned into pCI-neo (Hedegaard *et al.*, 2012). Mutant GluN1-Y647C, GluN1-R659T, GluN1-N674I, GluN1-D789N and GluN1-R794Q were generated by site-directed mutagenesis using the QuikChange^™^ protocol with Pfu DNA polymerase (Agilent Technologies). The parental strand was replicated with the desired mismatch incorporated into the primer (Yuan *et al.*, 2005). Methylated parental DNA template was digested with Dpn I. The nicked double-stranded mutant DNA was transformed into TOP10 Competent Cells (Life Technologies). The mutations were verified by sequencing through the region of the mutations. For whole-cell voltage clamp recordings the cDNA for human wild-type *GRIN1* and *GRIN2B* (Myc-DDK-tagged, based on GenBank accession codes NM_007327 and NM_000834, respectively) were subcloned into pCMV6-Entry (OriGene Technologies, catalogue numbers RC216458 and RC223623). Mutant GluN1-N674I was generated in pCMV6-GluN1 by site-directed mutagenesis using the QuikChange^™^ mutagenesis kit as described above and verified by Sanger sequencing.

### Two-electrode voltage clamp recordings

Two-electrode voltage clamp recordings were performed as previously described (Hansen *et al.*, 2013; Yuan *et al.*, 2014; Chen *et al.*, 2017*b*). Briefly, coding RNA for wild-type and mutant GluN1 was synthesized *in vitro* from linearized template cDNA and injected into *Xenopus laevis* oocytes (Ecocyte). Following injection, the oocytes were stored at 15–19°C in Barth’s solution containing (in mM) 88 NaCl, 2.4 NaHCO_3_, 1 KCl, 0.33 Ca(NO_3_)_2_, 0.41 CaCl_2_, 0.82 MgSO_4_ and 5 Tris/HCl (pH 7.4 with NaOH). Voltage-clamp recordings were performed 2–4 days post-injection at room temperature (23°C). The recording solution contained (in mM) 90 NaCl, 1 KCl, 10 HEPES, 0.5 BaCl_2_ and 0.01 EDTA (pH 7.4 with NaOH). Voltage and current electrodes were filled with 0.3 and 3.0 M KCl, respectively, and current responses were recorded at a holding potential of −40 mV (unless otherwise stated). Data acquisition and voltage control were accomplished with a two-electrode voltage-clamp amplifier (OC725, Warner Instrument). NMDA receptor agonists (glutamate or glycine) and antagonists (Mg^2+^ or H^+^) were applied to the oocyte using a computer-controlled eight-modular valve positioner (Digital MVP Valve, Hamilton). Glutamate (100 μM) and glycine (100 μM) were used in all oocyte experiments unless otherwise stated. The agonist concentration-response curves were fitted with:

(1) $$Response\left(\%\right)=\text{1}00/\left(\text{1}+{\left({\text{EC}}_{\text{5}0}/\left[agonist\right]\right)}^{nH}\right)$$

where EC_50_ is the agonist concentration that elicited a half-maximal response and *nH* is the Hill slope. IC_50_ values for Mg^2+^ were obtained by fitting the concentration-response data with:

(2) $$Response\left(\%\right)=\left(\text{1}00-minimum\right)/\left(\text{1}+{\left(\left[modulator\right]/{\text{IC}}_{\text{5}0}\right)}^{nH}\right)+minimum$$

where IC_50_ is the concentration that produces a half-maximal effect, and *minimum* is the degree of residual inhibition at a saturating concentration of Mg^2+^. Data were expressed as mean ± standard error of the mean (SEM).

### Preparation and transfection of HEK 293 cells

HEK 293 cells (ATCC) were plated onto glass coverslips coated in 100 µg/ml poly-d-lysine and incubated at 37°C (5% CO_2_ in DMEM/F12 1:1 media) supplemented with 10% foetal bovine serum, 2 mM glutamine, 50 U/ml penicillin and 50 µg/ml streptomycin. The cells were co-transfected with plasmid cDNAs encoding green fluorescent protein (GFP), GluN2B, and GluN1 or GluN1–N674I. For transfection, media was changed to S-MEM media with 1% foetal bovine serum, 0.5 mM glutamine and 0.5 mM CaCl_2_. Mixed pCMV plasmid DNA (0.25 μg each of GluN2B, GluN1 or GluN1-N674I, and 0.125 μg nucGFP) with 50 μl Opti-MEM^™^ and 1 μl Lipofectamine^™^ 2000 (Life Technologies) was added to each well and the mixture incubated at 37°C for 4 h. Media was then changed to transfection media plus 100 μM D-AP5 (R&D systems), a competitive NMDA receptor antagonist. The cells were inspected by fluorescence microscope 16–24 h post-transfection. Cells expressing GFP were used for whole-cell voltage-clamp recordings.

### Whole-cell voltage clamp recordings

Whole-cell voltage clamp recordings were performed as previously described (Jiang *et al.*, 2005; Thompson *et al.*, 2012). Membrane potential of transfected cells were held at −60 mV (at room temperature, 23°C) using a patch clamp amplifier (EPC10, HEKA). Borosilicate glass microelectrodes, 5–5.5 MΩ were filled with intracellular solution containing (in mM) 117 KCl, 10 NaCl, 11 HEPES, 11 EGTA, 2 MgCl_2_, 1 CaCl_2_, 2 Na_2_ATP (pH 7.2 with KOH). The extracellular solution contained (in mM) 135 NaCl, 5 KCl, 5 HEPES, 10 glucose, 1.2 MgCl_2_ and 1.25 CaCl_2_ (pH 7.4 with NaOH). Solution exchange was achieved with a rapid solution changer (BioLogic) and data collected using PatchMaster software (HEKA). Peak currents were defined as the maximal amplitude of response during the agonist application; responses were plotted as current density (pA/pF). Concentration-response curves were constructed and fitted by Equation 1 using Prism 7.

## Results

### *GRIN1* mutations in patients with polymicrogyria

**Figure 1:**
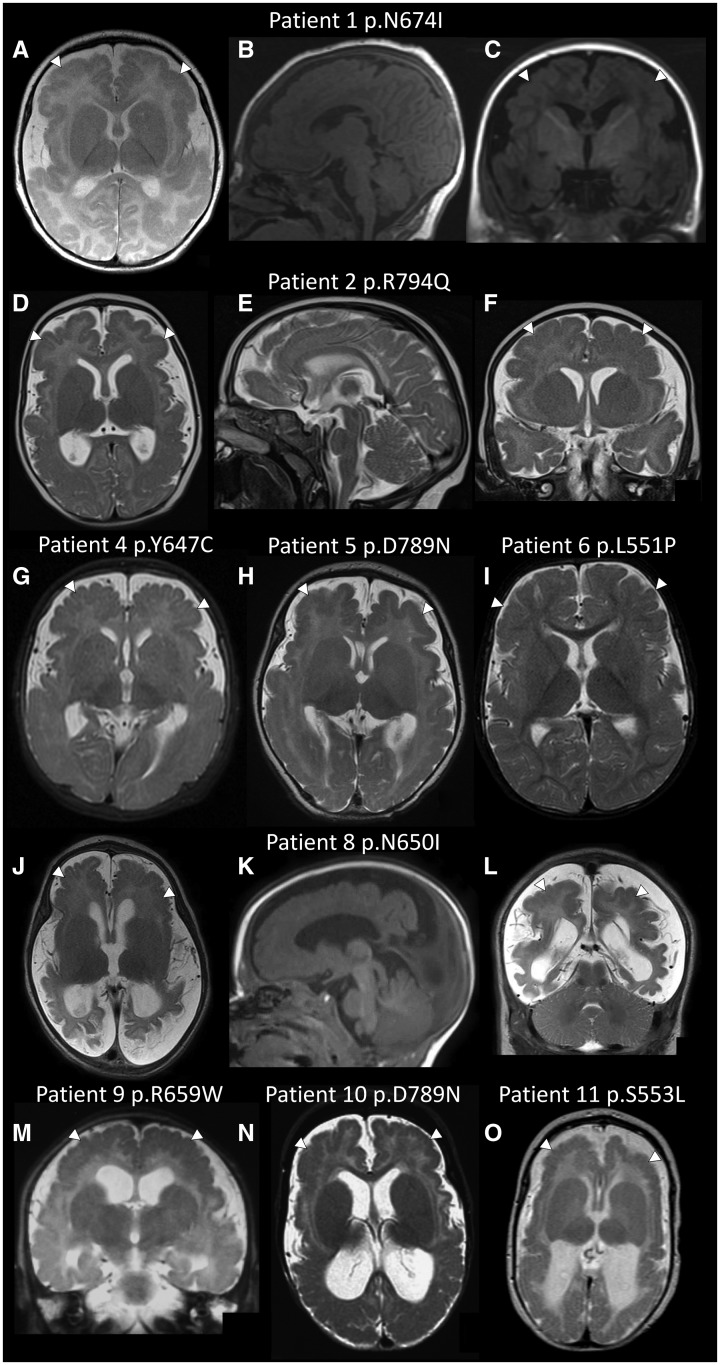
**Polymicrogyria in patients with *GRIN1* mutations.** Axial, midline sagittal and coronal brain magnetic resonance images for Patient 1 at age 2 months (**A–C**) and Patient 2 at age 5 months (**D–F**); axial magnetic resonance images for Patient 4 at age 3 months (**G**), Patient 5 at age 6 weeks (**H**) and Patient 6 at age 8 months (**I**); axial, sagittal and coronal images for Patient 8 at age 3 months (**J–L**); a coronal image for Patient 9 at age 4 months (**M**); axial images from Patient 10 at age 8 months (**N**) and Patient 11 at age 2 months (**O**). Images **B, C** and **K** are T_1_-weighted. All other images are T_2_-weighted. The images demonstrate bilateral extensive polymicrogyria (white arrows) more severe anteriorly. Note the increased extra-axial spaces and enlarged lateral ventricles (in most images apart from **I**) suggesting cerebral volume loss.

We performed whole-exome sequencing in 57 unrelated individuals with polymicrogyria and their unaffected parents. Two of the polymicrogyria patients (Patients 1 and 2) had *de novo* missense mutations in *GRIN1* [c.2021A>T, p.(Asn674Ile) and c.2381G>A, p.(Arg794Gln)]. Apart from known polymicrogyria genes, *GRIN1* was the only gene with *de novo* mutations in more than one patient. (A manuscript describing all *de novo* mutations in the cohort is in preparation). Given the predicted rate of *de novo* missense mutations in *GRIN1* (from ExAC: 4.4 × 10^−5^) the observation of more than one *de novo GRIN1* missense mutation in 57 subjects has a *P*-value of 3.1 × 10^−6^ (binomial test). This is close to a strict Bonferroni-corrected experiment-wide *P*-value threshold of 2.5 × 10^−6^ per gene (Kiezun *et al.*, 2012). We found nine further *GRIN1* missense mutations in additional MCD patients (Table 1). The 11 missense mutations were all *de novo*, affected highly-conserved residues (Supplementary Fig. 1) and *in silico* predictions suggested they were all deleterious (Supplementary Table 2). Two of the mutations [c.1975C>T, p.(Arg659Trp) and c.2365G>A, p.(Asp789Asn)] were recurrent. Shared clinical features in the live-born patients were severe or profound developmental delay, postnatal microcephaly, cortical visual impairment and treatment-resistant epilepsy. A summary of the clinical features is given in Table 1 (detailed case reports are provided in the Supplementary material). Some previous *GRIN1* patients have been noted to have abnormal eye movements resembling oculogyric crises and stereotypic hand movements. At least four of our series had similar features (abnormal movements in Patient 4, stereotypic movements in Patient 6, and episodes of gaze deviation in Patients 8 and 10). Abnormal movements may have been present in the other patients but either unreported or misinterpreted (e.g. as seizures or roving eye movements). Magnetic resonance images from all subjects (apart from Patients 3 and 7) were available for review (Fig. 1). The patients demonstrated an extensive bilateral cortical malformation similar in appearance to tubulinopathy- or *GRIN2B-*associated dysgyria (Platzer *et al.*, 2017). None of the brains have been examined histologically but the magnetic resonance appearance was most consistent with polymicrogyria. The distribution of the polymicrogyria was typically diffuse (frontal, perisylvian, parietal and temporal) and bilateral but with some occipital sparing. Patients 8 and 9 had polymicrogyria in the frontal and parietal regions but the extent of perisylvian involvement was unclear from the available images. Patient 6 had predominantly perisylvian polymicrogyria with some frontal, parietal and temporal involvement (grade 2 bilateral perisylvian polymicrogyria). This milder cortical malformation correlated with the patient’s milder phenotype (sitting and walking, but still severely delayed). Additional magnetic resonance findings in the patients were increased extra-axial spaces (particularly anteriorly, 9/11), enlarged lateral ventricles (8/11), reduced white matter volume (9/11), thinning of the corpus callosum (4/11) and abnormal hippocampi (3/11).

**Table 1:** Clinical features of patients with *GRIN1* mutations and polymicrogyria

Patient	1	2	3	4	5	6	7	8	9	10	11
Sex	Male	Female	Female	Male	Male	Female	Male	Female	Male	Male	Female
Age at last review	9 y 2 m	2 y 5 m	4 y 7 m	19 m	3 y 6 m	9 y	22 w gestation	20 m	Died at 14 y	17 y	Died at 8 y
Mutation	c.2021A>T	c.2381G>A	c.1975C>T	c.1940A>G	c.2365G>A	c.1652T>C	c.1958C>G	c.1949A>T	c.1975C>T	c.2365G>A	c.1658C>T
	p.(Asn674Ile)	p.(Arg794Gln)	p.(Arg659Trp)	p.(Tyr647Cys)	p.(Asp789Asn)	p.(Leu551Pro)	p.(Ala653Gly)	p.(Asn650Ile)	p.(Arg659Trp)	p.(Asp789Asn)	p.(Ser553Leu)
Birth OFC	n/a	n/a	+0.0 SD	−0.9 SD	−2.5 SD	−0.8 SD	n/a	+0.59 SD	n/a	−4.9 SD	n/a
Last OFC	−3.6 SD	−5.2 SD	−7.1 SD	−1.5 SD	−6.5 SD	n/a	−1.6 SD	−5.7 SD	n/a	−6.7 SD at 16 m	−7.1 SD at 3 y
Development	Profound delay. Not sitting or walking. Vocalizing.	Severe delay. Good head control. No babbling.	Profound delay. Not sitting or walking. No babbling.	Severe delay. Not sitting. Abnormal movements.	Profound delay. No motor or speech development.	Severe delay. Sitting. Walks with frame. Stereotypies.	n/a	Severe delay. Not sitting or walking. No babbling.	Severe delay. Not sitting or walking.	Severe delay. Not sitting or walking. Vocalizing.	Severe delay. Not sitting or walking.
CVI	Yes	Yes	Yes	Yes	Yes	Yes	n/a	Yes	n/a	Yes	Yes
Seizure onset	6 w	9 m	2 m	3 m	1 w	<1 y	n/a	<1 m	5 w	? <1 m	2 w
Initial seizure type	Myoclonic	Generalized tonic-clonic	Spasms	Tonic	Grimacing	Spasms	n/a	Tonic, gaze deviation	Tonic	Gaze deviation from 3 y	Tonic
Neurology	Spastic tetraplegia, axial hypotonia	Spastic tetraplegia, axial hypotonia	Spastic tetraplegia, axial hypotonia	Pseudobulbar palsy, hypotonia	Spastic tetraplegia, axial hypotonia	Spastic tetraplegia, axial hypotonia	n/a	Spastic tetraplegia, axial hypotonia	Mild scoliosis	Spastic tetraplegia, axial hypotonia	Spastic tetraplegia, axial hypotonia
Cortex	Extensive bilateral PMG with occipital sparing	Extensive bilateral PMG with occipital sparing	Diffuse bilateral PMG.	Extensive bilateral PMG with occipital sparing	Extensive bilateral PMG with occipital sparing	Bilateral perisylvian PMG, frontal, parietal and temporal spread	Abnormal thinning and sulcation of the cerebral cortex	Fronto-parietal PMG	Extensive bilateral PMG with occipital sparing	Diffuse bilateral PMG	Diffuse bilateral PMG
Corpus callosum	Normal	Normal	Thin	Normal	Normal	Normal	Hypoplastic	Thin	Normal	Normal	Thin
Lateral ventricles	Normal	Mildly enlarged	n/a	Mildly enlarged	Mildly enlarged	Normal	Large	Large	Moderately enlarged	Enlarged	Enlarged
Hippocampi	Normal	Normal	n/a	Normal	Abnormal	Normal	Normal	Abnormal	Normal	Normal	Thin leaves

Ages: weeks (w); months (m) and years (y); CVI = cortical visual impairment; n/a = not available/applicable; OFC = occipital frontal circumference; PMG = polymicrogyria.

Lemke *et al.* (2016) reviewed the MRI findings of 19 previous *GRIN1* patients. None were noted to have polymicrogyria (delayed formation of sulci was observed in one patient with homozygous *GRIN1* nonsense mutations) (Lemke *et al.*, 2016). To ensure our findings were not simply due to differences in the interpretation of radiology we reviewed MRI brain images from four previous non-polymicrogyria patients with *GRIN1* mutations (p.Asp552Glu, p.Met641Ile, p.Gly815Arg and p.Gly827Arg) (Ohba *et al.*, 2015; Lemke *et al.*, 2016). This confirmed the absence of polymicrogyria.

### Clustering of polymicrogyria-associated *GRIN1* mutations

**Figure 2:**
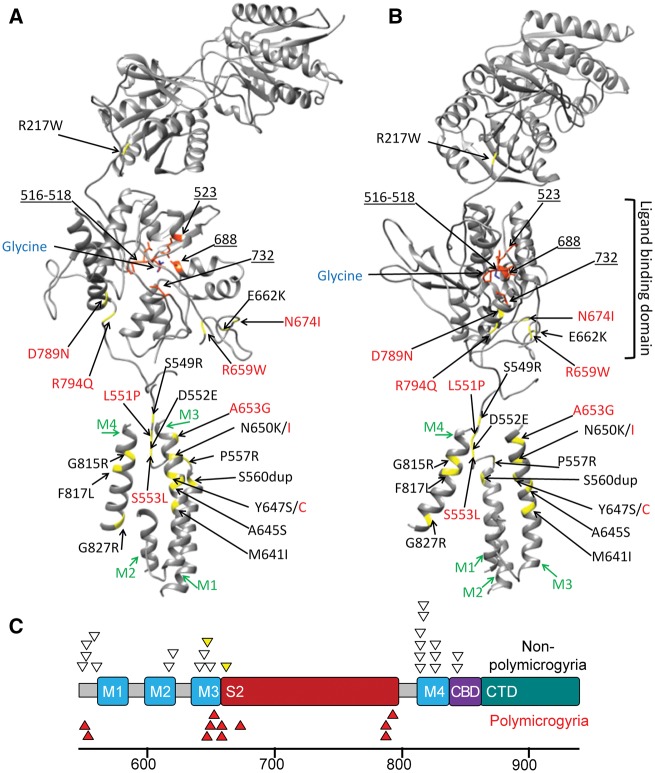
**Position of the *GRIN1* mutations.** (**A**) This ribbon diagram of the GluN1 subunit of the NMDA receptor demonstrates the location of the binding site of glycine in the ligand binding domain, transmembrane helices (M1-M4 in green) and glycine binding residues of GluN1 (orange residues and blue underlined numbers). Mutated residues are in yellow. Polymicrogyria-associated mutations are in red text and previous *GRIN1* mutations are in black text. (**B**) The same ribbon model rotated 90 degrees in the axial plane. (**C**) A model of the C-terminal end of *GRIN1.* In addition to showing M1-M4, the model shows the position of the second ligand binding domain (S2), calmodulin biding domain (CBD) and the C-terminal domain (CTD). Codon position is listed below the model. Heterozygous *GRIN1* variants reported or reviewed by Lemke *et al.* (2016) (non-polymicrogyria, white triangles) are shown above the model. The two yellow triangles represent subjects with CT brain imaging only. Polymicrogyria-associated *GRIN1* mutations (red triangles) cluster in the S2 domain (6/11) or in the adjacent Lurcher motif of M3 (3/11).

We compared the positions of the 11 polymicrogyria-associated *GRIN1* mutations with 16 different heterozygous proven (or likely) *de novo GRIN1* mutations previously reported in 23 patients with non-syndromic intellectual disability and epileptic encephalopathy (Fig. 2) (Lemke *et al.*, 2016). We observed that most polymicrogyria-associated mutations occurred in the S2 domain of GluN1 or the adjacent M3 helix. The proportion of mutation in these two domains (9/11) was greater than expected based on the distribution of previous *GRIN1* mutations (5/23; *P* = 0.002, Fisher’s exact test). Furthermore, no polymicrogyria-associated mutations were observed in M4, a domain where nearly half of all previous mutations were located.

Six polymicrogyria-associated *GRIN1* mutations [p.Arg659Trp (two mutations), p.Asn674Ile, p.Asp789Asn (two mutations) and p.Arg794Gln] were located in the S2 domain. The S2 domain forms part of the ligand-binding domain of GluN1 and has been shown to be highly intolerant to variation (Ogden *et al.*, 2017). The recurrent p.Arg659Trp mutation is in the DRPEER motif of S2 (residues 658–663). This motif is close to the extracellular end of M3, near the channel pore entrance. Only two previous *GRIN1* mutations have been located in S2 (p.Glu662Lys and p.Ser688Tyr) (Hamdan *et al.*, 2011; Zehavi *et al.*, 2017). Neither patient was reported to have polymicrogyria although the p.Glu662Lys patient only had a CT brain scan (MRI was not done) (Hamdan *et al.*, 2011). CT generally lacks the resolution to detect polymicrogyria.

Three polymicrogyria-associated *GRIN1* mutations (p.Tyr647Cys, Asn650Ile and p.Ala653Gly) were in close proximity to the S2 domain in a region of highly-conserved residues at the extracellular end of M3. These residues (646–654) are known as the Lurcher motif (SYTANLAAF). This motif is thought to act as the major permeation barrier at the intersection of four M3 helices (Murthy *et al.*, 2012). Different mutations affecting residues 647 and 650 have previously been reported in patients with epileptic encephalopathy (Epi4K Consortium *et al.*, 2013; Ohba *et al.*, 2015). Neither was reported to have polymicrogyria although the p.Tyr647Ser patient only had a CT brain scan. The final two polymicrogyria-associated mutations (p.Leu551Pro and p.Ser553Leu) were located in the S1-M1 linker region. The tertiary structure of GluN1 means the residues are located close to the extracellular end of the M3 helix (Fig. 2). It has recently been proposed that the pre-M1 region is close enough to M3 to interact as a key gating element (Sobolevsky *et al.*, 2009; Chen *et al.*, 2017*b*; Ogden *et al.*, 2017).

### Modelling the structural effects of *GRIN1* mutations

We hypothesized that polymicrogyria-associated and previous *GRIN1* mutations might have different effects on the structure of GluN1. To study this we developed a 3D model of the GluN1/GluN2A NMDA receptor based on the rat GluN1/GluN2B tetramer (Fig. 2). We simulated models for the nine polymicrogyria-associated *GRIN1* mutations and 16 previous *GRIN1* mutations. We measured each mutant model in three ways: (i) the displacement, between mutant and wild-type, of the extracellular and intracellular ends of each transmembrane helix; (ii) the displacement, between the mutant and wild-type, of nine key domains. A RMSD value (a measure of average distance between the two superimposed structures) was calculated for each domain; and (iii) the number of hydrogen bonds between the glycine ligand and the glycine-binding residues of GluN1.

Analysis of transmembrane helix positions (Supplementary Table 3) and domain-specific RMSD values (Supplementary Table 4) revealed no consistent differences between previous and polymicrogyria-associated *GRIN1* mutations. The regions of GluN1 most affected by *GRIN1* mutations in both groups were M2, M3 (particularly the extracellular end) and S2. All *GRIN1* mutants had RMSD values >2 Å for the S2 domain and DRPEER motif. The extracellular end of the M3 helix (the part of the helix involved in the channel pore entrance) was displaced >1 Å for all *GRIN1* mutants apart from polymicrogyria-associated p.Asp789Asn (0.67 Å). The p.Asp789Asn mutation stood out from other mutations in causing the least displacement of transmembrane helices (all ≤1.01 Å) and the lowest RMSD value for the M3 helix (1.17 Å). The p.Asp789Asn mutation still had its greatest effects on the S2 domain (RMSD 3.06 Å) where it was located. The polymicrogyria-associated p.Leu551Pro and p.Ser553Leu mutations were located in the S1-M1 linker region but had their greatest effects on the RMSD values of the M2, M3 and S2 domains. On average polymicrogyria-associated mutations generated slightly more hydrogen bonds with glycine (6.8) compared with previous mutations (4.8; *P* = 0.02, Mann-Whitney U-test) and wild-type (4) (Table 2). However, the effects varied between mutations. All polymicrogyria-associated mutations increased the number of hydrogen bonds with glycine apart from p.Asp789Asn, which formed one less than wild-type.

**Table 2:** Effect of *GRIN1* mutations on hydrogen bonds between the glycine ligand and the glycine-binding residues of GluN1

GluN1 residue	Pro516	Thr518	Arg523	Ser687	Ser688	Asp732	Bond total
**Wild-type**	−	××	×	−	×	−	4
**Previous *GRIN1* mutations**							
S549R	−	−	××	×	×	×	5
D552E	−	−	××	−	×	×	4
P557R	−	×	−	−	×	×	3
S560dup	−	×	×	−	×(689)	×(733)	4
G618R	−	−	−	−	××	×	3
G620R	−	×××	−	−	×	×	5
M641I	−	××	××	×	×	××	8
A645S	−	×	××	−	×	−	4
Y647S	−	−	−	×	××	××	5
N650K	−	−	−	×	×	×	3
E662K	−	×	×	−	×	×	4
G815R	−	×	×	−	××	×	5
G815V	−	×××	×××	−	×	×	8
F817L	−	−	−	−	××	×	3
G827R	−	××	××	−	×	×	6
R844C	−	××	××	−	×	×	6
**Polymicrogyria-associated *GRIN1* mutations**							
L551P	×	×××	×	−	××	××	9
S553L	−	××	××	××	−	−	6
Y647C	−	××	×	×	×	×	6
N650I	−	××	×	×	××	×	7
A653G	−	×××	×××	−	×	××	9
R659W	−	××	×	−	××	×	6
N674I	−	×××	×××	−	××	××	10
D789N	−	−	−	−	××	×	3
R794Q	−	−	−	×	××	××	5

Number of hydrogen bonds between glycine and specified residues: − (none), ×(one), ×× (two), ××× (three). All predicted hydrogen bonds were <3 Å in length.

### Impact of *GRIN1* mutations on NMDA receptor function

To investigate whether polymicrogyria-associated *GRIN1* mutations influence NMDA receptor function *in vitro* we undertook site-directed mutagenesis to introduce five of the *GRIN1* mutations (p.Tyr647Cys, p.Arg659Trp, p.Asn674Ile, p.Asp789Asn and p.Arg794Gln) into cDNA encoding human GluN1. We then expressed wild-type and mutant GluN1 with either human wild-type GluN2A or GluN2B in *Xenopus* oocytes and evaluated the effects of these mutants on pharmacological properties of NMDA receptors by using two-electrode voltage clamp recordings, including agonist potency (glutamate and glycine), as well as magnesium and proton sensitivity. The concentration that produced a half-maximal current response (EC_50_) was determined by measuring the current response to a range of glutamate (in the presence of 100 µM glycine) and glycine (in the presence of 100 µM glutamate) concentrations at a holding potential of −40 mV. The magnesium sensitivity (IC_50_) was determined by measuring the effect of different magnesium concentrations on agonist-evoked currents (by 100 µM glutamate and 100 µM glycine) at a holding potential of −60 mV. The proton sensitivity was evaluated as the percentage of receptor activity at pH 6.8 compared with receptor activity at pH 7.6 (holding potential of −40 mV).

The p.Arg659Trp and p.Arg794Gln mutations had similar effects; both increased the potency (reduced EC_50_ values) of agonists, and in particular, glutamate. The EC_50_ of mutant receptors was reduced to 10–20% of wild-type levels (Table 3). The potency of glycine was also increased: EC_50_ was 71% of wild-type for GluN1-R794Q/GluN2A, 61% for GluN1-R794Q/GluN2B, 36% for GluN1-R659W/GluN2A and 15% for GluN1-R659W/GluN2B. There were no detectable differences in Mg^2+^ blockade. Proton inhibition was not statistically significant apart from a reduced block in GluN1-R659W/GluN2A (70% mutant versus 52% wild-type).

**Table 3:** Summary of two-electrode voltage clamp data

Constructs	Glu EC_50_, µM (*n*)	Mutant/ WT, %	Gly EC_50_, µM (*n*)	Mutant/ WT, %	Mg^2+^ IC_50_, µM (*n*)	Mutant/ WT, %	%, pH 6.8/ pH 7.6	%, Mutant/WT
WT GluN1/GluN2A	3.3 ± 0.04 (6)		1.3 ± 0.05 (6)		24 ± 5.9 (5)		51 ± 1.7 (6)	
GluN1-R794Q/GluN2A	0.68 ± 0.06 (6)*	21	0.92 ± 0.09 (6)*	71	31 ± 5.4 (6)	129	47 ± 0.9 (6)	92
WT GluN1/GluN2B	1.7 ± 0.12 (7)		0.33 ± 0.05 (6)		18 ± 0.53 (6)		16 ± 0.6 (5)	
GluN1-R794Q//GluN2B	0.21 ± 0.03 (14)*	12	0.20 ± 0.02 (12)*	61	17 ± 1.7 (7)	94	17 ± 1.0 (7)	106
WT GluN1/GluN2A	3.2 ± 0.04 (6)		1.3 ± 0.01 (7)		24 ± 5.9 (5)		48 ± 6.4 (5)	
GluN1-N674I/GluN2A	2.2 ± 0.14 (6)	69	1.6 ± 0.08 (6)	123	34 ± 5.3 (6)	142	72 ± 3.2 (6)*	150
WT GluN1/GluN2B	1.5 ± 0.17 (10)		0.38 ± 0.06 (6)		26 ± 3.7 (8)		16 ± 0.9 (6)	
GluN1-N674I/GluN2B	1.9 ± 0.15 (14)	126	0.86 ± 0.05 (11)*	226	24 ± 2.6 (6)	92	50 ± 1.3 (6)*	313
WT GluN1/GluN2A	3.9 ± 0.28 (10)		1.0 ± 0.11 (11)		29 ± 4.1 (10)		52 ± 1.1 (12)	
GluN1-R659W/GluN2A	0.43 ± 0.03 (18)*	11	0.36 ± 0.09 (10)*	36	37 ± 3.5 (7)	128	70 ± 1.6 (11)*	135
WT GluN1/GluN2B	1.3 ± 0.09 (9)		0.33 ± 0.03 (12)		18 ± 0.5 (6)		16 ± 0.8 (10)	
GluN1-R659W/GluN2B	0.25 ± 0.04 (11)*	19	0.05 ± 0.01 (14)*	15	20 ± 3.4 (6)	111	15 ± 1.7 (10)	94
WT GluN1/GluN2A	3.8 ± 0.51 (6)		1.3 ± 0.06 (9)		24 ± 3.1 (6)		41 ± 2.0 (6)	
GluN1-Y647C/GluN2A	0.06 ± 0.03 (5)*	1.6	0.06 ± 0.01 (6)*	4.6	9.0 ± 1.0 (5)*	38	35 ± 2.1 (6)*	85
WT GluN1/GluN2B	1.7 ± 0.08 (7)		0.41 ± 0.05 (6)		21 ± 3.1 (5)		15 ± 2.5 (6)	
GluN1-Y647C/GluN2B	0.04 ± 0.01 (6)*	2.4	0.046 ± 0.015 (9)*	11	17 ± 4.3 (6)	81	30 ± 3.5 (6)*	200

Data were from two-electrode voltage-clamp recordings on *Xenopus* oocytes at −40 mV holding potential (except for Mg^2+^ at −60 mV) and expressed as mean ± SEM (*n*); WT = wild-type; *P < 0.05, unpaired *t*-test, compared to the corresponding data from wild-type receptors recorded on the same day.

The p.Tyr647Cys mutation demonstrated a profound increase in the potency of glutamate with an EC_50_ of just 1.6% of wild-type for GluN1-Y647C/GluN2A and 2.4% for GluN1-Y647C/GluN2B. There was a similarly increased sensitivity to glycine (EC_50_ 4.6% for GluN1-Y647C/GluN2A; 11.2% for GluN1-Y647C/GluN2B). There was a statistically significant increase in Mg^2+^ blockade for GluN1-Y647C/GluN2A (IC_50_ 38% of wild-type) but not GluN1-Y647C/GluN2B (IC_50_ 81% of wild-type) (Table 3). There were also significant but variable effects on proton inhibition: slightly increased in GluN1-Y647C/GluN2A (35% mutant versus 41% wild-type) but reduced in GluN1-Y647C/GluN2B (30% mutant versus 15% wild-type).

**Figure 3:**
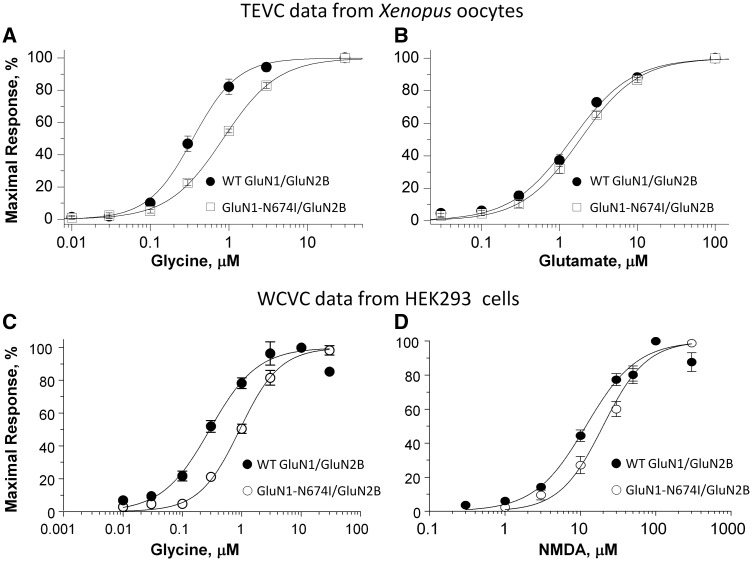
**The p.Asn674Ile mutation changes the response of the NMDA receptor to agonists.** The *top* graphs display concentration-response curves for (**A**) glycine (in the presence of 100 µM glutamate) and (**B**) glutamate (in the presence of 100 µM glycine) determined by two-electrode voltage-clamp (TEVC) recordings from *Xenopus* oocytes expressing either wild-type (WT)-GluN1/GluN2B or GluN1-N674I /GluN2B. The *bottom* graphs display concentration-response curves for (**C**) glycine (in the presence of 100 µM NMDA) and (**D**) NMDA (in the presence of 100 µM glycine) determined by whole-cell voltage-clamp (WCVC) recordings from transfected HEK 293 cells expressing either WT-GluN1/GluN2B or GluN1-N674I /GluN2B. Error bars represent SEM.

The p.Asn674Ile mutant had a different profile of effects. The EC_50_ for glycine was unchanged for GluN1-N674I/GluN2A but increased to 226% for GluN1-N674I/GluN2B (Table 3 and Fig. 3A). This indicated a reduced potency for glycine. The EC_50_ of glutamate was not significantly altered for GluN1-N674I/GluN2A or GluN1-N674I/GluN2B (Table 3 and Fig. 3B). However, p.Asn674Ile did demonstrate significantly reduced proton inhibition (72% GluN1-N674I/GluN2A versus 48% wild-type GluN1/GluN2A; 50% GluN1-N674I/GluN2B versus 16% wild-type GluN1/GluN2B). There was no significant difference in Mg^2+^ blockade for p.Asn674Ile receptors. To confirm that p.Asn674Ile reduced glycine potency we assessed the mutation in a second, separate *in vitro* heterologous expression setting. We co-expressed wild-type and mutant GluN1 with human GluN2B in HEK 293 cells and evaluated the concentration-effect curves for agonist (NMDA) and co-agonist (glycine) using whole-cell voltage clamp. The whole-cell voltage clamp results (Fig. 3C and D) were consistent with the two-electrode voltage clamp findings and demonstrated a pronounced loss of sensitivity to glycine (EC_50_ 310% of wild-type; GluN1-N674I/Glu2B: 0.93 µM, *n* = 8; versus wild-type GluN1/Glu2B: 0.30 µM, *n* = 8; *P* < 0.0001; unpaired *t*-test) and a moderate reduction in the potency of NMDA (EC_50_ 164% of wild-type; GluN1-N674I/Glu2B: 19.71 µM, *n* = 6; versus wild-type GluN1/Glu2B: 12.05 µM, *n* = 8; *P* = 0.01; unpaired *t*-test).

Multiple attempts (mutagenesis, RNA syntheses, RNA injection, and recordings) were made to express p.Asp789Asn mutant receptors (as both GluN1/GluN2A and GluN1/GluN2B) in *Xenopus* oocytes. The current amplitudes at saturating agonist concentrations (up to 1 mM glutamate and 3 mM glycine at holding potential of −40 mV) were too small to characterize the effects on NMDA receptor pharmacological properties (GluN1-D789N/Glu2A: 13 ± 2.8 nA, *n* = 14; versus wild-type GluN1/Glu2A: 855 ± 199 nA, *n* = 14; GluN1-D789N/Glu2B: 11 ± 3.1 nA, *n* = 14; versus wild-type GluN1/Glu2B: 585 ± 156 nA, *n* = 14). We therefore evaluated the expression levels of GluN1-D789N using a β-lactamase activity assay in transiently-transfected HEK cells (Supplementary material) (Swanger *et al.*, 2016). The ratio of surface-to-total protein levels for the mutant GluN1-D789N was reduced to 71 ± 4.2 % (*n* = 4; *P* = 0.04; unpaired *t*-test) of wild-type when co-expressed with wild-type GluN2A, and had no significant change (115 ± 46 %; *n* = 8; *P* = 0.46; unpaired *t*-test) when co-expressed with wild-type GluN2B (Supplementary Fig. 2), suggesting that the complete loss of current responses for GluN1-D789N is not due to a trafficking defect or lack of surface expression but rather a functional change in the membrane-bound receptor.

## Discussion

By using exome data from parent-offspring trios we identified 2 of 57 polymicrogyria patients with *de novo* missense mutations in *GRIN1*. Another nine *de novo* missense *GRIN1* mutations were identified in additional MCD patients. Shared features in the patients were extensive bilateral polymicrogyria, severe or profound developmental delay, postnatal microcephaly, cortical visual impairment and treatment-resistant epilepsy. *De novo* missense mutations in *GRIN1* have previously been reported in patients with non-syndromic intellectual disability (Hamdan *et al.*, 2011; Redin *et al.*, 2014; Zhu *et al.*, 2015; Lemke *et al.*, 2016; Rossi *et al.*, 2017), movement disorders (Ohba *et al.*, 2015; Chen *et al.*, 2017*a*; Zehavi *et al.*, 2017), epileptic encephalopathy (Epi4K Consortium *et al.*, 2013), and cerebral visual impairment (Bosch *et al.*, 2016). The MRI features seen in previous *GRIN1* patients were non-specific volume loss and generalized cerebral atrophy but not polymicrogyria (Lemke *et al.*, 2016). We note that at least two previous *GRIN1* patients only had CT brain scans, which cannot reliably detect polymicrogyria. This raises the possibility that some previous *GRIN1* patients may have had unrecognized polymicrogyria. It is also possible that apparently non-polymicrogyria *GRIN1* patients (by MRI) may have subtle structural brain abnormalities that are below the resolution of current scanning technology.

The NMDA receptor is a tetrameric heteromultimer that comprises two GluN1 subunits (encoded by *GRIN1*) and two variable GluN2 subunits, which are encoded by *GRIN2A, GRIN2B, GRIN2C* or *GRIN2D*. Like GluN1, GluN2B is extensively expressed in the cerebral cortex during foetal development (Liu *et al.*, 2004). *GRIN2B* mutations have been found in patients with autism (O’Roak *et al.*, 2011; Tarabeux *et al.*, 2011), cerebral visual impairment (Bosch *et al.*, 2016), West syndrome (Lemke *et al.*, 2014) and intellectual disability (Endele *et al.*, 2010; Adams *et al.*, 2014; Lemke *et al.*, 2014; Hu *et al.*, 2016; Swanger *et al.*, 2016). *GRIN2B* mutations have also recently been observed in MCD patients (Platzer *et al.*, 2017). In contrast to the foetus, the main GluN2 subunit of the adult brain is GluN2A. This change occurs during early postnatal development and is thought to be a key developmental switch (Liu *et al.*, 2004). *GRIN2A* mutations have been found in patients with epilepsy-aphasia spectrum disorders (Carvill *et al.*, 2013; Lemke *et al.*, 2013; Lesca *et al.*, 2013; Gao *et al.*, 2017), early-onset epileptic encephalopathy (Endele *et al.*, 2010; Pierson *et al.*, 2014; Yuan *et al.*, 2014; Swanger *et al.*, 2016) and schizophrenia (Tarabeux *et al.*, 2011). However, consistent with its mainly postnatal expression, *GRIN2A* mutations have not been reported to cause MCDs.

The reason why some *GRIN1* patients get polymicrogyria is uncertain. This may require additional factors in the patient’s genetic background or environmental conditions during gestation. Only two of our series were known to have had testing for cytomegalovirus. Patient 5 had negative postnatal serology. Patient 6 had positive testing (urine PCR and serum IgM) at 4 months of age. However, his mother had negative serology at 36 weeks, which suggests the infection occurred postnatally. Other patients were not tested because they lacked typical features of cytomegalovirus infection (e.g. rash, retinitis, brain calcification or deafness). It has not been possible to retrospectively screen the other patients for cytomegalovirus. We observed that polymicrogyria-associated *GRIN1* mutations clustered in the S2 or M3 domains, regions that are significantly depleted of variation in control populations (Swanger *et al.*, 2016; Ogden *et al.*, 2017). In addition, the GluN1 S2 domain has rarely been mutated in non-polymicrogyria *GRIN1* patients. The GluN1 S2 domain is critical to the binding of glycine, the co-agonist of the NMDA receptor. Therefore, the observation of this putative genotype–phenotype correlation suggests that polymicrogyria and non-polymicrogyria mutations may have different effects on co-agonist binding or the activation state of the receptor. Inspecting the 3D model of GluN1 suggests the polymicrogyria-associated mutations in S2 reside close to the extracellular end of the M3 transmembrane helix that forms a bundle crossing that occludes ion permeation. These variants may therefore alter gating. The polymicrogyria-associated p.Arg659Trp mutation was located in the DRPEER motif of S2. This motif is a highly charged motif thought to influence the relative permeability of Ca^2+^ ions through the channel (Watanabe *et al.*, 2002). Similarly, the three M3 mutations (p.Tyr647Cys, p.Asn650Ile and p.Ala653Gly) had milder effects on the wider structure of the receptor but were located in the highly-conserved Lurcher motif, which controls NMDA receptor gating.

Our structural modelling found polymicrogyria-associated mutations were associated with an increase in the average number of hydrogen bonds between the glycine ligand and the glycine-binding residues of GluN1. The formation of additional hydrogen bonds may alter the kinetics of co-agonist binding (e.g. increased affinity). No other stark differences between polymicrogyria-associated and previous *GRIN1* mutations were observed. The measurements used (transmembrane helix position and domain-specific RMSD) may not have been sensitive to key differences between the two groups. In addition, the structural modelling suggests there is heterogeneity in how polymicrogyria-associated mutations affect GluN1 structure. This may have confounded comparison between the two groups.

Two-electrode voltage clamp analysis showed that three of the polymicrogyria-associated mutations (p.Tyr647Cys, p.Arg659Trp and p.Arg794Gln) significantly increased the potency of both glutamate and glycine. This increased potency may mean that mutant receptors can be activated at lower concentrations of agonist than wild-type receptor. Several lines of evidence link excess NMDA receptor signalling to polymicrogyria. The intracerebral injection of ibotenate has been used for decades to generate *in vivo* models of epilepsy and cortical malformations including polymicrogyria (Marret *et al.*, 1996; Takano *et al.*, 2004). Ibotenate is an agonist of both NMDA and glutamatergic metabotropic receptors. Intracerebral ibotenate injection in newborn mice (Marret *et al.*, 1995), hamsters (Marret *et al.*, 1996; Takano *et al.*, 2004) and rats (Takano and Matsui, 2015) causes a range of grey and white matter changes, including polymicrogyria-like lesions, similar to those seen following perinatal hypoxic/ischaemic insults. Over-stimulation of NMDA receptors is thought to lead to excitotoxicity due to excess calcium influx through the receptor channel (Choi *et al.*, 1988; Zhou *et al.*, 2013). Profound gain-of-function NMDA receptor subunit mutations are excitotoxic when expressed *in vitro* (Li *et al.*, 2016; Ogden *et al.*, 2017). NMDA receptor-related excitotoxicity has been implicated in hypoxic/ischaemic events (Simon *et al.*, 1984; Rothman and Olney, 1986). Hypoxic/ischaemic events during foetal brain development are a well-recognized cause of polymicrogyria in humans. Calcium influx through the NMDA receptor can lead to activation of a range of cellular pathways including the pro-survival PI3K-AKT pathway (Lafon-Cazal *et al.*, 2002; Wang *et al.*, 2012). Activating mutations in components of the PI3K-AKT pathway have been found in polymicrogyria patients (Riviere *et al.*, 2012; Mirzaa *et al.*, 2014). MCDs including polymicrogyria are a feature of Zellweger syndrome, a rare metabolic disorder caused by peroxisomal dysfunction. Analysis of a mouse model of Zellweger syndrome showed that the neuronal migration abnormalities seen in the mice were due to NMDA receptor-mediated calcium mobilization (Gressens *et al.*, 2000). Another metabolic disorder associated with polymicrogyria is glycine encephalopathy (Dobyns, 1989). The hyperglycinaemia in this condition may enhance the excitotoxic activity of glutamate acting through the NMDA receptor (Subramanian *et al.*, 2015).

In contrast to the three potential gain-of-function polymicrogyria-associated *GRIN1* mutations, most previous *GRIN1* mutations caused dominant-negative effects resulting in a significant loss of receptor function (Lemke *et al.*, 2016). Animal models suggest that NMDA receptor hypofunction is less likely to disturb gross cortical structure. Mice homozygous for GluN1 null alleles die soon after birth from respiratory problems but do not have severe abnormalities of neuronal migration (Messersmith *et al.*, 1997). Similarly, mice with a GluN1 deletion limited to excitatory cortical neurons have only subtle disturbance of cortical structure (Iwasato *et al.*, 2000). However, a simple model of gain-of-function *GRIN1* mutations causing polymicrogyria is challenged by the results for p.Asn674Ile. Both two-electrode and whole-cell voltage clamp analyses were consistent in showing this mutation decreased the potency of glycine and possibly (to a lesser extent) glutamate. How can we explain this apparently paradoxical finding? There are a number of possible mechanisms that might explain why p.Asn674Ile causes polymicrogyria.

First, the p.Asn674Ile mutation caused a significant loss of proton inhibition. GluN2B receptors are inhibited with an IC_50_ at physiological pH. Therefore loss of proton inhibition will potentiate responses even at resting pH. This may mean neurons are prone to excess calcium influx, a problem that will be exacerbated in low pH environments (e.g. during oxidative stress). Recent work has shown the importance of the balance of signalling through synaptic and extra-synaptic NMDA receptors (Zhou *et al.*, 2015). Activation of synaptic NMDA receptors activates pro-survival pathways. In contrast, massive and prolonged co-activation of both synaptic and extrasynaptic receptors leads to cell death. If p.Asn674Ile blunts the response of synaptic receptors (e.g. through reduced agonist potency) while promoting signalling at extrasynaptic receptors (e.g. through reduced proton inhibition) this may tip the balance in some neurons towards apoptosis. Alternatively, polymicrogyria may be a consequence of disturbed NMDA receptor signalling regardless of whether there is gain- or loss-of-function. There is evidence from *in vitro* studies that a low-level background of NMDA activation is needed to support neuronal survival (Zhou *et al.*, 2015), guide radial migration (Behar *et al.*, 1999; Hirai *et al.*, 1999) and promote neuronal differentiation (Yoneyama *et al.*, 2008). Transient delivery of an NMDA antagonist to a focal area of the cortex of newborn rats disturbs cortical lamination and generates heterotopic cell clusters (Reiprich *et al.*, 2005). A range of NMDA antagonists, including ethanol, have been shown to induce apoptosis in the brains of developing rats (Olney *et al.*, 2002). This is of relevance as polymicrogyria has occasionally been reported in patients with foetal alcohol syndrome (Reinhardt *et al.*, 2010). Finally, it remains possible that p.Asn674Ile (and other polymicrogyria-associated *GRIN1* mutations) may have additional effects that have not been captured by our electrophysiological analysis.

If polymicrogyria-associated *GRIN1* mutations mainly cause gain of function while non-polymicrogyria mutations cause loss of function it raises the question why the two types cause such similar phenotypes (severe developmental delay, spasticity, early onset seizures, postnatal microcephaly, cerebral visual impairment and stereotypic movements). A potential explanation is that gain-of-function mutations may cause cell death (due to excitotoxicity) soon after neurons begin expressing NMDA receptors. Early cell loss would thin the foetal cortex (leading to polymicrogyria) and result in a postnatal cortex depleted of cells expressing NMDA receptors. In contrast, loss-of-function mutations may cause cell death more gradually (e.g. due to loss of NMDA-mediated pro-survival signalling) missing the key window when polymicrogyria occurs (<22 weeks). Having cells in the postnatal cortex which are insensitive to glutamate (due to loss-of-function mutations) may be functionally equivalent to the cells being absent (due to gain-of-function mutations). The evidence of cerebral atrophy observed in several patients was present in infancy and progressed on subsequent scans (Patient 11). The atrophy is likely due to the direct effects of mutations (excitotoxicity or loss of pro-survival signalling) as well as damage from frequent seizures.

In conclusion, we have found *de novo GRIN1* missense mutations in patients with extensive bilateral polymicrogyria. Our results provide evidence for a genotype–phenotype correlation with most polymicrogyria-associated *GRIN1* mutations clustering in the S2 or M3 domains, regions of the protein rarely mutated in non-polymicrogyria patients or the normal population. In addition, we showed that polymicrogyria-associated *GRIN1* mutations significantly alter *in vitro* NMDA receptor function. Our results confirm the importance of *de novo* mutations in the aetiology of MCDs and polymicrogyria; expand the phenotypic spectrum associated with *GRIN1* mutations; demonstrate similarities between human polymicrogyria and animal models of the disorder; and highlight the important role of NMDA signalling in the pathogenesis of polymicrogyria.

## Supplementary Material

Supplementary Data

## Abbreviations

EC_50_: concentration of agonist required for half-maximal effect
IC_50_: concentration of antagonist required for half-maximal inhibition
MCD: malformation of cortical development
NMDA: *N*-methyl-d-aspartate
RMSD: root-mean-square deviation
