# Supplementary material for: De novo mutations in GRIN1 cause extensive bilateral polymicrogyria
Source: Brain. 2018 Jan 22;141(3):698–712. doi: 10.1093/brain/awx358 (PMC5837214; doi:10.1093/brain/awx358)
Supplement: Supplementary Data [file awx358_brain-2017-01315-file005.pdf]

***De novo* mutations in *GRIN1* cause extensive bilateral polymicrogyria.**

Fry AE *et al*.

Supplementary Material

Contents **Page**

Case reports	Detailed clinical descriptions of the eleven patients with *GRIN1* mutations.	2
Supplementary Methods	Additional details of the methods used for exome sequencing and β-lactamase activity assay.	11
Supplementary Figure 1	Sequences from orthologs and paralogs of *GRIN1* demonstrating conservation of the mutated residues.	14
Supplementary Figure 2	β-lactamase activity assay measuring NMDA receptor surface expression	15
Supplementary Table 1	Demographic and phenotypic details for the cohort of 57 UK polymicrogyria patients who were exome sequenced.	17
Supplementary Table 2	Molecular details and *in silico* predictions for the *GRIN1* mutations found in the polymicrogyria patients.	18
Supplementary Table 3	The effect of *GRIN1* mutations on the positions of the four transmembrane helices of GluN1.	19
Supplementary Table 4	Root-mean-square deviation measurements for domains of GluN1 for each *GRIN1* mutation.	21
Supplementary Table 5	Molecular details and *in silico* predictions for the additional *de novo* mutations found in Patients 1-4.	23

Case reports

Patient 1

This male patient was the first child of non-consanguineous White British parents. There was no family history of learning disability, epilepsy or polymicrogyria. He had two healthy younger sisters. The patient was born by normal vaginal delivery at 37 weeks gestation following an uncomplicated pregnancy. Birth weight was 2.95kg (+0.1 standard deviations [SD]). There were no early concerns about head size or muscle tone. The patient was discharged on day 1 but fed poorly and returned to hospital for assessment on day 3. Feeding (by breast and bottle) subsequently improved. The patient began having myoclonic seizures at 6 weeks. The patient was last reviewed at 9 years and 2 months of age. He had profound developmental delay. He could not sit independently due to low truncal tone and had no speech but could vocalise when happy. His response to stimuli was inconsistent, but he demonstrated some ability to express preference (eye pointing or switch pressing). The patient would smile or giggle when moved or bounced. He would become frustrated if not stimulated and could express anger. Reflux was a frequent problem until a gastrostomy tube was inserted (at 8 years of age). He was mainly gastrostomy fed due to an unsafe swallow. He also had bilateral Achilles tendon release due to limb spasticity. The patient's epilepsy continued despite trying a range of medications (>12). A ketogenic diet helped partially. Vagal nerve stimulator was being considered. Seizures included at least four types: frequent myoclonic jerks, daily vacant episodes, focal motor seizures (2-3/day), and occasional generalised tonic-clonic seizures. The patient also had nocturnal episodes of breath holding associated with loud vocalisation ('hooting') on expiration. On examination the patient had microcephaly (occipitofrontal circumference [OFC] 48.5cm, -3.6 SD). Weight was 24.6kg (-1.1 SD) and length 128cm (-1.0 SD). He had roving eye movement, strabismus, severe spastic quadriplegia and axial hypotonia. The patient had a distinctive face (partly due to hypotonia of the facial musculature) with a broad nasal root and small epicanthic folds. The patient had small puffy hands and feet. MRI brain scan at 2

months of age showed extensive frontal, perisylvian, parietal and temporal polymicrogyria with occipital sparing. Corpus callosum, cerebellum and brain stem were normal. There was no evidence of congenital infection. Extensive metabolic investigation, routine karyotype, array comparative genome hybridization (array CGH) and sequencing of the *GPR56* gene were all normal.

Patient 2

This female patient was the second child of non-consanguineous British parents of Pakistani heritage. There was no family history of learning disability, epilepsy or polymicrogyria. She was born by normal vaginal delivery at 39+5 weeks gestation following an uncomplicated pregnancy. Birth weight was 3.4kg (+0.0 SD). The patient was noted to be an irritable infant. She initially breast fed well but was admitted for dehydration at 1 month of age. Feeding was changed to bottle and improved. At 4 months she was found to have microcephaly, visual inattentiveness, increased limb tone and clenched fists. The patient's first seizure was at 9 months and was associated with fever. The patient was diagnosed with cortical visual impairment. She was noted to have pale optic discs. Visual evoked potential testing showed absent responses to flash/pattern stimulation. At 10 months her length was 69.2cm (-1.0 SD), weight 9.18kg (+0.3 SD) and head circumference 41.2cm (-3.9 SD). The patient went on to have severe developmental delay. The patient was last reviewed at 2 years and 5 months of age. She was able to smile and laugh but was unable to sit or roll. She had gradually become more visually responsive. She could look toward light and respond to sound. There were no concerns about her hearing. The patient struggled with feeding. She was eating only pureed food and had severe dribbling. Constipation was an intermittent problem. The patient had infrequent generalised tonic-clonic seizures associated with grunting and screaming, often triggered by fever or teething. She was not on any anti-epileptic medication. On examination the patient had an OFC of 43.4cm (-5.2 SD). She had spastic quadriplegia with clenched hands. Her plantar reflexes were upgoing but she was not particularly hyperreflexic. She had axial hypotonia but head control was reasonable. There was no fixing and following. She had deep set eyes, long palpebral

fissures, small upturned nose, thin upper lip, small chin and an excoriated rash around her neck (due to the dribbling). MRI at 5 months of age showed extensive polymicrogyria with occipital sparing. The lateral ventricles were enlarged and the periventricular white matter was reduced. Corpus callosum and posterior fossa were normal. Metabolic investigation, TORCH screen, routine karyotype and array CGH were all normal. It is unknown if the TORCH screen included testing for cytomegalovirus.

Patient 3

This female patient was the child of non-consanguineous French parents. The pregnancy was complicated by maternal diabetes. The patient was born by caesarean section at 38+5 weeks gestation. She had transient respiratory distress following delivery. Her birth weight was 2.8kg (-0.9 SD), length 49cm (-0.2 SD) and OFC 34cm (0.0 SD). There were no early feeding problems reported. She presented at 2 months of age with infantile spasms. The patient was last reviewed at 4 years and 7 months of age. She had profound developmental delay. There was no history of regression. The patient was unable to sit unsupported and had no language development. She had cortical visual impairment. There were no concerns about hearing. She was gastrostomy fed and had problems with severe dribbling and intermittent constipation. Seizure types include frequent myoclonic jerks, daily spasms, and occasional tonic-clonic seizures. On examination, the patient had an OFC of 43 cm (-7.1 SD), height was 93 cm (-3.0 SD) and weight was 15.4 kg (-1.0 SD). She had spastic quadriplegia with axial hypotonia, severe dribbling and strabismus. MRI brain scan was reported to show diffuse bilateral polymicrogyria with thin corpus callosum. Basal ganglia, brainstem and cerebellum were normal. Array CGH and sequencing of *GPR56* and *TUBB2B* were normal.

Patient 4

This male patient was the child of non-consanguineous French parents. He was born by caesarean section at 40 weeks gestation. Birth weight was 3.58kg (+0.1 SD), length 49cm (-0.2 SD) and OFC 34cm (-0.9 SD). Concerns were raised in the neonatal period because of irritability, frequent crying and episodes of hyperextension. There were no early feeding problems. Tonic seizures began at 3 months of age. The patient was last reviewed at 19 months of age. He had severe developmental delay. He was not sitting and had no language development. The patient had weekly spasms, cortical visual impairment, problems with constipation and was gastrostomy fed. On examination his OFC was 47.5cm (-1.5 SD), length 79cm (-1.3 SD) and weight 15kg (+2.4 SD). He had spastic quadriplegia, axial hypotonia and roving eye movements. He was not considered to be dysmorphic. An MRI brain scan at 3 months of age showed extensive polymicrogyria with occipital sparing. Extensive metabolic investigation was normal.

Patient 5

This male patient was the child of non-consanguineous Israeli parents. He was born by normal vaginal delivery at 40+2 weeks gestation following an uncomplicated pregnancy. Birth weight was 3.37kg (-0.4 SD). His head size was noted to be small at birth (OFC 32cm, -2.5 SD). In the first week of life he developed episodes of facial grimacing, screaming and hand fisting. These were considered to be seizures. Drug therapy (phenobarbital followed by vigabatrin, topiramate, clobazam and lamotrigine) was initially ineffective. Ketogenic diet and steroid treatment helped reduce seizure frequency. The patient was last reviewed at 3 years and 6 months of age. He had profound developmental delay with no motor or speech development. He had severe cortical visual impairment (no fixing and following, blinking only in a dark room when a direct light beam was close to his eyes). In contrast he responded promptly, usually by smiling, to noise or tactile stimuli. He was fed pureed food orally. The patient had been seizure free for around 18 months on a mixture of clobazam, levetiracetam and prednisolone. On examination he had severe microcephaly (OFC

42.5cm, -6.5 SD), spastic quadriplegia, axial hypotonia and roving eye movements. Height was 87cm (-3.1 SD) and weight 13.5kg (-1.3 SD). MRI brain scan at 6 weeks of age showed extensive bilateral polymicrogyria affecting the frontal, parietal, and temporal lobes. There was delayed myelination and mildly enlarged lateral ventricles. Underdevelopment of the hippocampi was noted. EEG initially showed left temporal spike activity with subsequent bilateral multifocal epileptic discharges. Metabolic investigation, cytomegalovirus testing and array CGH were all normal.

Patient 6

This female patient was the child of non-consanguineous Israeli parents. There was no family history of learning disability, epilepsy or polymicrogyria. She was born at term by normal vaginal delivery following an uncomplicated pregnancy. Birth OFC was 33.5cm (-0.8 SD). The patient presented in the first year of life with infantile spasms. The patient was last reviewed at 9 years of age. She had severe developmental delay and was being educated in a special needs school. The patient could sit unsupported and walk with a walking frame. She had no speech but could communication through a tablet computer using her left arm. The patient had some behavioural problems with severe tantrums and autistic features. It was not clear if she smiled socially. Seizures were controlled with sodium valproate. She had oromotor problems but was still orally fed. On examination the patient had spastic quadriplegia, strabismus, pseudobulbar palsy and stereotypic movements. MRI brain scan at 8 months of age showed bilateral perisylvian polymicrogyria extending into the posterior frontal, anterior parietal and temporal lobes. Array CGH was normal. Urine PCR and serum IgM were positive for cytomegalovirus at age 4 months, but her mother was IgG and IgM negative at 36 weeks.

Patient 7

Fetal ultrasound found this single live male fetus had moderate ventriculomegaly (15 mm), abnormal thinning and sulcation of the cerebral cortex, dysplasic corpus callosum, small head size

(5th percentile, -1.6 SD) and shortened long bones (2nd-5th percentile). The fetus had clenched hands, left clubbed foot and a right rocker bottom foot. The fetus was 22 weeks gestation by dates but only 20 weeks and 6 days based by size. Fetal brain MRI showed severe parenchymal thinning throughout both cerebral hemispheres, with moderate to severe ventriculomegaly. There was abnormal cortical infolding along the left frontal lobe. There was a hypoplastic corpus callosum and mildly enlarged cerebellum. The brainstem appeared normal. The pregnancy was terminated. The parents declined fetal autopsy. The parents were non-consanguineous and had a mixed European heritage. There was no family history of brain malformations. The fetus had a normal array CGH.

Patient 8

This female patient was the child of non-consanguineous parents of mixed European heritage. There was no family history of learning disability, epilepsy or polymicrogyria. Clubfeet were noted on prenatal ultrasound at 22 weeks gestation. She was born at 32+6 weeks gestation by caesarean section. Birth weight was 624 g (-4.7 SD), length 36 (-4.9 SD) and OFC 31 cm (+0.59 SD). Seizures began in the neonatal period with gaze deviation, tonic stiffening and desaturations. Severe developmental delay was noted in the first months of life. The patient was last reviewed at 20 months of age. She was not sitting yet and had no language development. The patient was gastrostomy fed. Her problems included cortical visual impairment, bilateral club feet, constipation, poor linear growth with excessive weight gain, chronic restrictive lung disease, secundum atrial septal defect and spina bifida occulta. The patient continued to have daily seizures associated with gaze deviation and tonic stiffening. On examination the patient had severe microcephaly (OFC 39cm [at 1 year 10 months], -5.7 SD), spastic quadriplegia, axial hypotonia and stereotypic movements. Length and weight (at 2 years 6 months) were 74 cm (-4 SD) and 13.81 kg (+0.53 SD) respectively. She had bitemporal narrowing, prominent midface, thick eyebrows, button nose, thick lips, and a high-arched palate. MRI brain scan at 3 months of age showed bilateral fronto-parietal polymicrogyria, diffuse cerebral volume loss, dilated lateral and third ventricles, enlarged extra-

axial spaces, diffuse abnormal white matter signal (likely secondary to delayed myelination), thin corpus callosum and abnormal hippocampi bilaterally with incomplete folding. Metabolic investigation and array CGH were both normal.

<u>Patient 9</u>

This male patient was the second child of non-consanguineous African American parents. There was a family history of epilepsy but not learning disability or polymicrogyria. He had one older and one younger brother who were healthy. He was born by normal vaginal delivery at 39 weeks gestation. He had frequent vomiting during the first weeks of life. His feeding was slow and associated with tongue biting and frequent aspiration. Seizures began at 5 weeks. He had tonic seizures (bunched up body, clenched fists and red in face), possibly infantile spasms, which were initially occurred individually but later clustered. Subsequently he had intractable seizures with myoclonic jerks and generalised tonic-clonic seizures (sometimes 30+/day). Medication tried included sodium valproate, prednisolone, lamotrigine and lorazepam. At the age of 9 he was given vigabatrin which seemed to help the best. The patient had severe developmental delay. He had a gastrostomy placed at age 8 years. On examination he had a mild scoliosis. He was not dysmorphic. The patient had severe respiratory problems with recurrent aspiration and pneumonias. He died at the age of 14 years. MRI brain scan at 4 months of age showed large extra-axial spaces with extensive frontal, perisylvian, parietal and temporal polymicrogyria. The posterior lateral ventricles were dysplastic and moderately enlarged. Corpus callosum, cerebellum and brain stem were normal. Array CGH was normal.

<u>Patient 10</u>

This male patient was the fifth child of non-consanguineous parents of mixed European heritage. There was a family history of epilepsy but not learning disability or polymicrogyria. He had five siblings (4 older, 1 younger) who were all well. The pregnancy was complicated by maternal

anaemia and a urinary tract infection for which his mother took an antibiotic. He was born by normal vaginal delivery at 36 weeks gestation. Birth weight was 2.64 kg (-0.1 SD), length 52.1 cm (+2.3 SD) and OFC 26.4 cm (-4.9 SD). There were concerns about his head size and possible seizures in the neonatal period. At 3 months of age he was noted to have unusual eye opening/closing and hardly ever opened his eyes. He smiled socially around 10 months. At 16 months of age his length was 69 cm (-4.0 SD), weight 8.7 kg (-2.3 SD) and OFC 40.5 cm (-6.7 SD). The patient was diagnosed with seizures at 3 years of age. Initially these took the form of spells of abnormal gaze (he would look upward and get stuck there) with little or no responsiveness during spells. The seizures were well controlled on medication (zonisamide). He continued to have occasional gelastic seizures during illnesses and other stressors. At 5 years of age he began having recurrent aspiration, vomiting and poor weight gain. He had a gastrostomy soon afterwards. The patient had severe developmental delay. By the age of 17 years he was not sitting unsupported yet. He had nystagmus, esotropia, and little visual tracking. His hearing was good and he made some vocalisations. On examination he had severe scoliosis, contractures of the hips, small penis, and truncal hypotonia with spastic quadriparesis. MRI brain scan at 8 months revealed diffuse bilateral polymicrogyria, mildly-enlarged extra axial spaces, reduced white matter and enlarged lateral ventricles. Basal ganglia, corpus callosum, cerebellum and brain stem were normal. Array CGH was normal.

Patient 11

This female patient was the only child of non-consanguineous White American parents. There was a family history of epilepsy but not learning disability or polymicrogyria. The pregnancy was complicated by hyperemesis and breech presentation. She was born by caesarean section at 39 weeks gestation. She fed poorly and had severe frequent vomiting during her first 6 months. She had a gastrostomy at 6 months of age. Fundoplication was performed at 1 year. Post-operative complications meant that she was unable to get enough nutrition without vomiting or retching. A

gastro-jejunal tube was tried but would not stay in. She had a Roux-en-Y jejunostomy at age 7 years. Seizures began at 2 weeks of age. Initial these included tonic episodes (stiff, apnoeic and blue) and generalized tonic clonic seizures. The seizures were continuous in the first year of life. At 2-3 years of age she was still having frequent daily seizures (10-50/day). The seizures were individually short, lasting less than 1 minute, but often occurred back to back in clusters lasting up to 5 minutes. The seizures were characterised by stiffness and staring but little jerking apart from a quick jump or startle at the start. At ages 5-7 years the 1-5 minute spells continued but she was also having longer more severe seizures associated with prolonged stiffness and apnoea, followed by clonic jerking, jaw clenching and teeth grinding. She also had prolonged post-ictal sleepiness (up to 24 hours). Persistent limb spasticity was noted at 18 months of age. She had a lot of back arching from early infancy although this was thought to be due to reflux. Her OFC at 3 years and 3 months was 42 cm (-7.1 SD). The patient had severe global developmental delay. She was able to smile socially but unable to sit unsupported. She had abnormal visual orienting behaviour possibly due to delayed visual maturation superimposed on cortical visual impairment. She had a neurogenic bladder. The patient had markedly increased seizures in the last year of life and died at the age of 8 years. MRI brain scan at 2 months showed marked microcephaly with diffuse bilateral polymicrogyria. There were mildly enlarged extra axial spaces, mildly thin leaves of the hippocampus, reduced white matter volume, enlarged lateral ventricles and thin corpus callosum. Basal ganglia, cerebellum and brain stem were normal. Repeat MRI brain at 3 years showed increased brain atrophy.

Supplementary Methods

Exome Sequencing

For Patients 1 and 2, genomic DNA was extracted from blood and sequenced using a Nextera Rapid Capture Expanded Exome kit (Illumina) followed by 100 bp paired-end sequencing on an Illumina HiSeq2000. Reads were mapped to the 1000 Genomes version of the human reference genome (human_g1k_v37) using the Burrows-Wheeler Aligner v0.7.5a (Li and Durbin, 2009). Single-nucleotide variants and indels were jointly called and genotyped using the Genome Analysis Tool Kit (GATK v3.3.0) (McKenna *et al.*, 2010) and annotated using SnpEff v3.3 and SnpSift (Cingolani *et al.*, 2012a; Cingolani *et al.*, 2012b). Variants were annotated for allele frequency using ExAC data (release 0.3, 14th January 2016) (Lek *et al.*, 2016). *De novo* mutations were identified using the GATK SelectVariants walker according to previously described criteria (Epi4K Consortium *et al.*, 2013). We excluded synonymous variants and variants with minor allele frequency >0.001. All putative *de novo* mutations were validated by Sanger sequencing in the proband and both parents. For Patients 3 and 4, library generation, enrichment, whole exome sequencing and variant calling were performed as previously described (Poirier *et al.*, 2013). Additional *de novo* mutations were found in subjects 1-4 (Supplementary Table 5). These were considered unlikely to explain the patients' phenotypes.

β-lactamase activity assay

The β-lactamase activity assay was performed as described previously (Swanger *et al.*, 2016). In brief, HEK 293 cells were seeded in 96-well plates and transiently transfected with pCI-neo plasmid with cDNA encoding β-lac-GluN1 or β-lac-GluN1-D789N using Fugene6 (Promega, Madison, WI). After 24 hours, cells were washed with Hank's Balanced Salt Solution (HBSS) supplemented with 10 mM HEPES, then the surface activity was measured by adding 100 µL of a 100 µM nitrocefin (Millipore, Billerica, MA) solution in HBSS with HEPES to the wells. To determine total activity, the cells were lysed in 50 µl H_2O for 30 min prior to the addition of 50 µl of 200 µM nitrocefin and

the absorbance was read at 468 nm every min for 30 min using a microplate reader at 30°C. All reagents were purchased from Sigma (unless otherwise stated). Data were expressed as mean ± standard error of the mean. Significance for all tests using unpaired *t*-test was set at $P < 0.05$.

References for the Supplementary Methods

Cingolani P, Patel VM, Coon M, Nguyen T, Land SJ, Ruden DM, et al. Using Drosophila melanogaster as a Model for Genotoxic Chemical Mutational Studies with a New Program, SnpSift. Front Genet 2012a; 3: 35.

Cingolani P, Platts A, Wang le L, Coon M, Nguyen T, Wang L, et al. A program for annotating and predicting the effects of single nucleotide polymorphisms, SnpEff: SNPs in the genome of Drosophila melanogaster strain w1118; iso-2; iso-3. Fly (Austin) 2012b; 6: 80-92.

Epi4K Consortium, Epilepsy Phenome/Genome Project, Allen AS, Berkovic SF, Cossette P, Delanty N, et al. De novo mutations in epileptic encephalopathies. Nature 2013; 501: 217-21.

Lek M, Karczewski KJ, Minikel EV, Samocha KE, Banks E, Fennell T, et al. Analysis of protein-coding genetic variation in 60,706 humans. Nature 2016; 536: 285-91.

Li H, Durbin R. Fast and accurate short read alignment with Burrows-Wheeler transform. Bioinformatics 2009; 25: 1754-60.

McKenna A, Hanna M, Banks E, Sivachenko A, Cibulskis K, Kernytsky A, et al. The Genome Analysis Toolkit: a MapReduce framework for analyzing next-generation DNA sequencing data. Genome Res 2010; 20: 1297-303.

Poirier K, Lebrun N, Broix L, Tian G, Saillour Y, Boscheron C, et al. Mutations in TUBG1, DYNC1H1, KIF5C and KIF2A cause malformations of cortical development and microcephaly. Nat Genet 2013; 45: 639-47.

Swanger SA, Chen W, Wells G, Burger PB, Tankovic A, Bhattacharya S, et al. Mechanistic Insight into NMDA Receptor Dysregulation by Rare Variants in the GluN2A and GluN2B Agonist Binding Domains. Am J Hum Genet 2016; 99: 1261-1280.

Species	Gene	Peptide ID	Protein sequence	
Homo sapiens	GRIN1	ENSP00000360608	540- ILVKK-EIPRSTLDSFMQPFQS-560	640- AMIIVASYTANLAAFLVLDRPEERITGINDPR--LRNPSDK----FI-680
Gorilla gorilla gorilla	GRIN1	ENSGGOP00000018945	NFLKQ-EIPRSTLDSFMQPFQS	AMIIVASYTANLAAFLVLDRPEERITGINDPR--LRNPSDK----FI
Canis lupus familiaris	GRIN1	ENSCAFP00000030425	ILVKK-EIPRSTLDSFMQPFQS	AMIIVASYTANLAAFLVLDRPEERITGINDPR--LRNPSDK----FI
Equus caballus	GRIN1	ENSECAP00000022237	ILVKK-EIPRSTLDSFMQPFQS	AMIIVASYTANLAAFLVLDRPEERITGINDPR--LRNPSD-----FI
Mus musculus	Grin1	ENSMUSP00000028335	ILVKK-EIPRSTLDSFMQPFQS	AMIIVASYTANLAAFLVLDRPEERITGINDPR--LRNPSDK----FI
Rattus norvegicus	Grin1	ENSRNOP00000029227	ILVKK-EIPRSTLDSFMQPFQS	AMIIVASYTANLAAFLVLDRPEERITGINDPR--LRNPSDK----FI
Gallus gallus	GRIN1	ENSGALP00000039568	ILVKK-EIPRSTLDSFMQPFQS	AMIIVASYTANLAAFLVLDRPEERITGINDPR--LRNPSDK----FI
Xenopus tropicalis	grin1	ENSXETP00000049601	ILVKK-EIPRSTLDSFMQPFQS	AMIIVASYTANLAAFLVLDRPEERITGINDPRTQLRNPSDK----FI
Danio rerio	grin1a	ENSDARP00000107703	ILVKK-EIPRSTLDSFMQPFQS	AMIIVASYTANLAAFLVLDRPEERITGINDPR--LRNPSDK----FI
Drosophila melanogaster	Nmdar1	FBpp0078410	ILEKK-PSRSSTLVSFLQPFSN	AMIIVASYTANLAAFLVLERPKTKLSGINDAR--LRNTMEN----LT
Homo sapiens	GRIN3A	ENSP00000355155	ILV-RTRDTAAPIGAFMWPLHW	CMFCLSTYTANLAAVMVGEKIYEELSGIHDPK--LHHPSQG----FR
Homo sapiens	GRIN3B	ENSP00000234389	IMV-RARDTASPIGAFMWPLHW	CLLVLSSYTANLAAVMVGDKTFEELSGIHDPK--LHHPAQG----FR
Homo sapiens	GRIN2A	ENSP00000379818	VMVSRSNGTVSPS-AFLEPFSA	AVIFLASYTANLAAFMIQEEFVDQVTGLSDKK--FQRPHD-YSPPFR
Homo sapiens	GRIN2B	ENSP00000477455	VMVSRSNGTVSPS-AFLEPFSA	AVIFLASYTANLAAFMIQEEYVDQVSGLSDKK--FQRPND-FSPPFR
Homo sapiens	GRIN2C	ENSP00000293190	VMVARSNGTVSPS-AFLEPYSP	AVIFLASYTANLAAFMIQEQYIDTVSGLSDKK--FQRPQD-QYPPFR
Homo sapiens	GRIN2D	ENSP00000263269	VMVARSNGTVSPS-AFLEPYSP	AVIFLASYTANLAAFMIQEEYVDTVSGLSDRK--FQRPQE-QYPPLK

Homo sapiens	GRIN1	ENSP00000360608	780- HE-NGFMEDL-DK-TWVRY----QECDS-800
Gorilla gorilla gorilla	GRIN1	ENSGGOP00000018945	HE-NGFMEDL-DK-TWVRY----QECDS
Canis lupus familiaris	GRIN1	ENSCAFP00000030425	HE-NGFMEDL-DK-TWVRY----QECDS
Equus caballus	GRIN1	ENSECAP00000022237	HE-N-FMENL-DKNVGCGT----KECDS
Mus musculus	Grin1	ENSMUSP00000028335	HE-NGFMEDL-DK-TWVRY----QECDS
Rattus norvegicus	Grin1	ENSRNOP00000029227	HE-NGFMEDL-DK-TWVRY----QECDS
Gallus gallus	GRIN1	ENSGALP00000039568	HE-NGFMEDL-DK-TWVRY----QECDS
Xenopus tropicalis	grin1	ENSXETP00000049601	HE-NGFMEEL-DK-TWVRY----QECDS
Danio rerio	grin1a	ENSDARP00000107703	HE-NGFMEDL-DK-TWVRY----QECDS
Drosophila melanogaster	Nmdar1	FBpp0078410	HE-SGFMEKL-DK-QWIFHGHVQQNCEL
Homo sapiens	GRIN3A	ENSP00000355155	KS-HGFMDMLHDK--WYRV----VPCGK
Homo sapiens	GRIN3B	ENSP00000234389	KS-SGFIDLLHDK--WYKM----VPCGK
Homo sapiens	GRIN2A	ENSP00000379818	VG-DGEMEEL-ET-LWLTG-----ICHN
Homo sapiens	GRIN2B	ENSP00000477455	FG-DGEMEEL-EA-LWLTG-----ICHN
Homo sapiens	GRIN2C	ENSP00000293190	LG-DGETQKL-ET-VWLSG-----ICQN
Homo sapiens	GRIN2D	ENSP00000263269	LGDD-EIEML-ER-LWLSG-----ICHN

Supplementary Figure 1. Sequences from orthologs and paralogs of *GRIN1* demonstrating conservation of the mutated residues. Protein alignments derived from Ensembl comparative genomics resources (http://www.ensembl.org/). Residues mutated in polymicrogyria patients are underlined and in red. Non-conserved residues are in light grey.

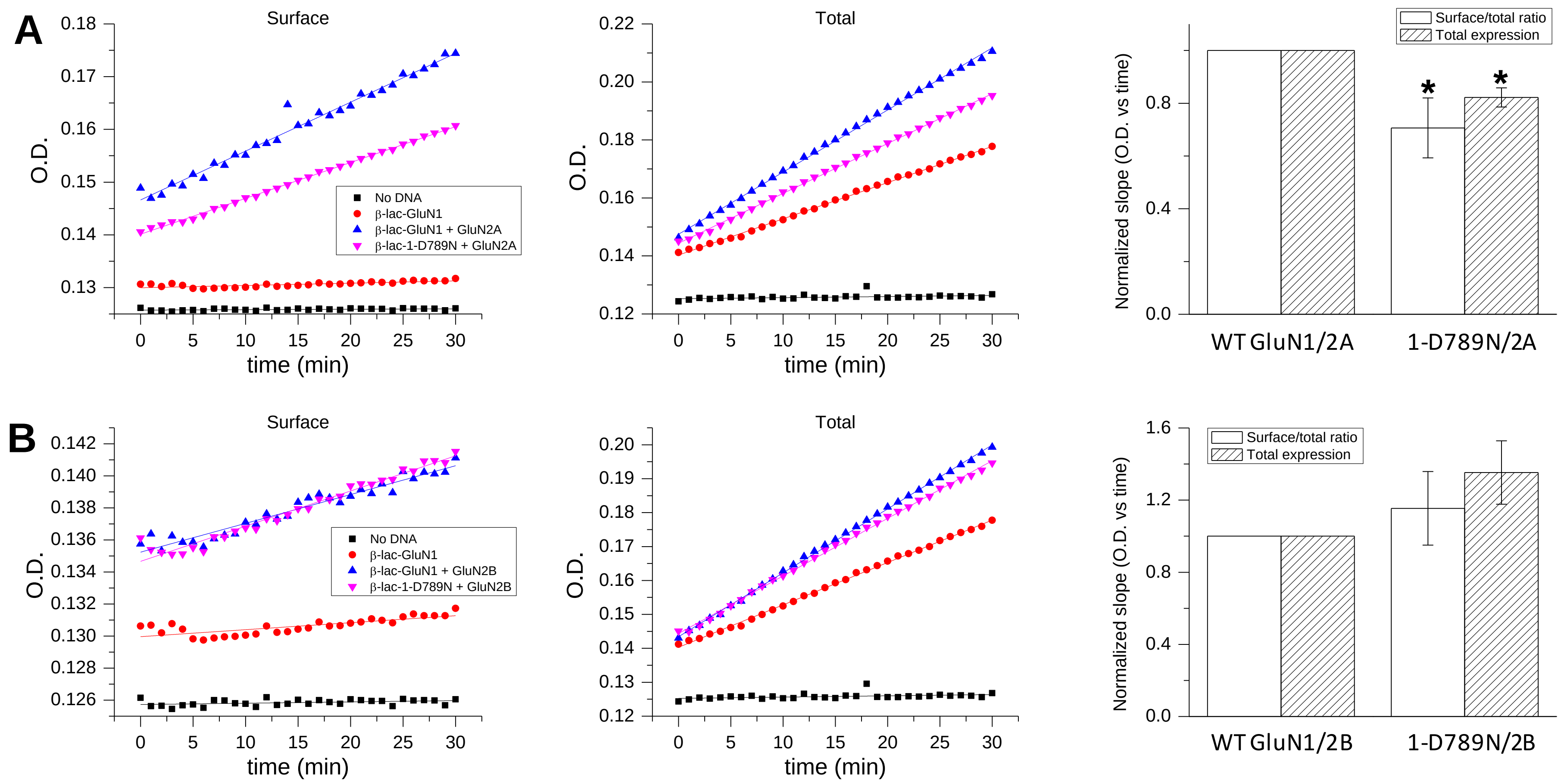

Supplementary Figure 2. β-lactamase activity assay measuring NMDA receptor surface expression.

β-lactamase activity was measured for HEK293 cells transiently transfected with (**A**) β-lac-GluN1+ GluN2A, β-lac-1-GluN1-D789N + GluN2A, β-lac-GluN1, or no DNA and (**B**) β-lac-GluN1+ GluN2B, β-lac-1-GluN1-D789N + GluN2B, β-lac-GluN1, or no DNA. Nitrocefin absorbance at 468 nm (optical density, O.D.) is plotted versus time for intact (surface expression, left graph) and lysed (total protein, middle graph) cells. The data were fit

with a straight line, and the slope of each absorbance versus time line was averaged across wells within each condition. The slope (mean ± SEM) for surface and total levels from 4 (for **A**) and 8 (for **B**) independent experiments are graphed on the right. * $P < 0.05$, unpaired t-test compared with the corresponding WT (wild type) receptors.

Supplementary Table 1. Demographic and phenotypic details for the cohort of 57 UK polymicrogyria patients who were exome sequenced.

Age at recruitment	<2 years	8
	2-5 years	25
	6-10 years	9
	11-15 years	6
	16+ years	9
Gender	Male 34 Female 23	
Ethnic origin	White British	46
	Other or mixed European	6
	Non-white	5
Parental consanguinity	No 54 Yes 3	
Polymicrogyria	Bilateral perisylvian	24
	Unilateral perisylvian	4
	Bilateral frontal or frontoparietal [a]	9
	Bilateral generalised [b]	8
	Unilateral generalised	2
	Bilateral parietal or parieto-occipital	6
	Unilateral parietal	3
	Bilateral temporal	1
Additional MRI features	Schizencephaly	9
	Periventricular heterotopia	7
Head size	Microcephaly (< -2 SD)	28
	Normocephaly (-2 to 0 SD)	19
	Normocephaly (0 to +2 SD)	7
	Macrocephaly (> +2 SD)	1
	Not known	2

Neurology	Spastic quadriplegia	21
	Right hemiplegia	4
	Left hemiplegia	6
	Diplegia	4
	Pseudobulbar palsy only	12
	Normal or not known	10
Motor delay	None	4
	Mild	18
	Moderate (e.g. walking >2 years)	11
	Severe (e.g. walking >5 years)	18
	Profound (minimal development)	6
Speech delay	None	13
	Mild	3
	Moderate (e.g. talking >2 years)	20
	Severe (e.g. talking >5 years)	15
	Not applicable	6
Oromotor dysfunction	None	10
	Mild	10
	Moderate (drooling, pureed food)	26
	Severe (gastrostomy fed)	11
Education	Mainstream school	7
	Mainstream class with support	12
	Special class in mainstream school	3
	Special School	18
	Not applicable	17
Epilepsy	Yes	44
	No	13

a With or without perisylvian or temporal involvement.
b With or without occipital sparing.

Supplementary Table 2. Molecular details and *in silico* predictions for the *GRIN1* mutations found in the polymicrogyria patients.

Patients	Gene	GenBank accession ID	Location	cDNA/ Protein	Inherit-ance	ExAC	PhyloP	Grantham	SIFT	Mutation taster	PolyPhen-2	Scaled CADD	M-CAP
6	*GRIN1*	NM_007327.3	Chr9: g.140056643 T>C	c.1652T>C p.(Leu551Pro)	*De novo*	-	3.68	98	Deleterious (0)	Disease causing (1)	Benign (0.059)	Top 1% (21.5)	Possibly Pathogenic (0.347)
11	*GRIN1*	NM_007327.3	Chr9: g.140056649 C>T	c.1658C>T p.(Ser553Leu)	*De novo*	-	4.48	145	Deleterious (0)	Disease causing (1)	Probably damaging (1)	Top 0.1% (32)	Possibly Pathogenic (0.425)
4	*GRIN1*	NM_007327.3	Chr9: g.140057118 A>G	c.1940A>G p.(Tyr647Cys)	*De novo*	-	2.79	194	Deleterious (0)	Disease causing (1)	Probably damaging (1)	Top 1% (25.4)	Possibly Pathogenic (0.821)
8	*GRIN1*	NM_007327.3	Chr9: g.140057127 A>T	c.1949A>T p.(Asn650Ile)	*De novo*	-	4.32	149	Deleterious (0)	Disease causing (1)	Benign (0.214)	Top 1% (29.7)	Possibly Pathogenic (0.758)
7	*GRIN1*	NM_007327.3	Chr9: g.140057136 C>G	c.1958C>G p.(Ala653Gly)	*De novo*	-	5.29	60	Deleterious (0)	Disease causing (1)	Probably damaging (0.999)	Top 1% (28.6)	Possibly Pathogenic (0.692)
3, 9	*GRIN1*	NM_007327.3	Chr9: g.140057153 C>T	c.1975C>T p.(Arg659Trp)	*De novo*	-	1.17	101	Deleterious (0)	Disease causing (1)	Probably damaging (1)	Top 0.1% (33)	Possibly Pathogenic (0.871)
1	*GRIN1*	NM_007327.3	Chr9: g.140057305 A>T	c.2021A>T p.(Asn674Ile)	*De novo*	-	4.32	149	Deleterious (0.01)	Disease causing (1)	Probably damaging (0.999)	Top 1% (28.6)	Possibly Pathogenic (0.580)
5, 10	*GRIN1*	NM_007327.3	Chr9: g.140058042 G>A	c.2365G>A p.(Asp789Asn)	*De novo*	-	4.64	23	Deleterious (0)	Disease causing (1)	Probably damaging (1)	Top 1% (29.9)	Possibly Pathogenic (0.168)
2	*GRIN1*	NM_007327.3	Chr9: g.140058058 G>A	c.2381G>A p.(Arg794Gln)	*De novo*	-	4.64	43	Tolerated (0.45)	Disease causing (1)	Possibly damaging (0.664)	Top 1% (25.5)	Possibly Pathogenic (0.054)

Supplementary Table 3. The effect of *GRIN1* mutations on the positions of the four transmembrane helices of GluN1.

Mutation	Domain	TM helix End Residue	M1 Top (extra) 559	M1 Tail (intra) 581	M2 Top 615	M2 Tail (intra) 606	M3 Top (extra) 655	M3 Tail (intra) 630	M4 Top (extra) 810	M4 Tail (intra) 828
Previous *GRIN1* mutations										
S549R	S1-M1		0.848	6.161	0.525	0.385	3.025	1.563	0.857	0.839
D552E	S1-M1		0.865	2.839	0.293	0.184	2.69	2.287	0.649	1.568
P557R	S1-M1		0.324	1.02	0.49	0.36	1.163	1.069	0.491	0.512
S560dup	M1		3.665	4.537	3.715	3.725	5.305	3.027	4.134	3.614
G618R	M2-M2		0.717	3.746	0.409	0.601	4.273	0.885	0.335	1.049
G620R	M2-M3		0.533	3.179	1.026	3.364	1.607	1.281	1.222	1.263
M641I	M3		0.97	3.318	0.229	0.194	4.34	0.373	0.53	1.342
A645S	M3		1.921	1.747	0.556	0.15	2.85	0.81	1.06	1.983
Y647S	M3 (sYtanlaaf)		0.736	1.731	0.522	0.711	2.644	0.937	0.674	0.53
N650K	M3 (sytaNlaaf)		1.969	2.655	3.265	1.172	2.578	0.839	1.052	4.026
E662K	S2 (drpeEr)		0.679	0.948	0.907	3.279	2.544	2.302	4.969	2.675
G815R	M4		0.238	2.819	1.052	3.532	1.418	1.495	0.904	1.738
G815V	M4		0.657	1.214	0.814	0.588	3.563	0.681	0.704	1.149
F817L	M4		1.12	1.406	3.315	0.896	1.892	1.548	1.986	2.731
G827R	M4		0.188	6.395	0.645	0.347	5.774	0.575	0.797	4.879
R844C	CBD		1.37	4.412	1.279	1.998	10.814	1.033	0.922	1.832
Polymicrogyria-associated *GRIN1* mutations										
L551P	S1-M1		2.333	1.761	0.898	1.083	6.505	2.98	1.556	1.768
S553L	S1-M1		0.682	0.471	1.474	1.504	1.402	1.152	0.632	1.833
Y647C	M3 (sYtanlaaf)		1.023	6.655	0.714	0.427	1.592	1.698	1.149	0.463
N650I	M3 (sytaNlaaf)		0.803	0.847	1.419	1.522	1.351	1.701	0.279	0.461
A653G	M3 (sytanlaAf)		3.129	2.058	0.924	3.38	2.797	2.851	1.308	0.286
R659W	S2 (dRpeer)		0.973	3.001	3.668	1.506	8.194	1	2.039	0.926
N674I	S2		1.618	3.228	0.911	3.439	8.557	1.856	3.418	2.456
D789N	S2		0.381	1.009	0.207	0.291	0.67	0.517	0.538	0.984
R794Q	S2		1.235	3.518	1.112	3.39	4.946	0.804	1.476	0.639
Mann–Whitney U test (*P*-value)			0.229	0.487	0.329	0.229	0.934	0.357	0.487	0.057

Distance (Å)	less than 1	1 - 2.999	3 - 4.999	5 - 9.999	10 or more

Distance (in Angstroms [Å]) between mutant and wild-type models calculated for specific residues (measured at the alpha carbon atom). Measurements were made for residues at both ends (top/tail) of each transmembrane (TM) helix (M1-M4). Extra(cellular) end; Intra(cellular) end; S1-2, first and second ligand binding domains; CBD, Ca^{2+}/calmodulin binding domain.

Supplementary Table 4. Root-mean-square deviation measurements for domains of GluN1 for each *GRIN1* mutation.

Mutation	Location	Domain Residues	AT 23-394	S1 395-544	M1 560-580	M2 606-615	M3 637-657	S2 658-808	M4 813-833	Average (AT to M4)	DRPEER 658-663	SYTANLA AF 646-654
Previous *GRIN1* mutations												
S549R	S1-M1		1.587	1.728	3.211	3.384	2.401	2.554	1.444	2.330	2.780	2.692
D552E	S1-M1		1.497	2.296	2.301	1.641	2.197	2.552	1.863	2.050	2.077	1.925
P557R	S1-M1		1.458	1.897	2.380	2.429	1.291	2.556	1.529	1.934	2.301	1.263
S560dup	M1		1.358	2.219	1.916	2.877	3.837	6.009	1.887	2.872	2.309	4.496
G618R	M2-M3		1.509	1.813	2.392	2.096	3.010	2.949	1.350	2.160	3.081	3.697
G620R	M2-M3		1.639	1.990	2.293	3.177	2.332	2.745	3.573	2.536	3.792	1.870
M641I	M3		1.513	1.983	2.239	1.854	3.002	2.669	1.973	2.176	3.088	0.860
A645S	M3		1.507	1.868	1.922	1.894	2.124	2.775	1.852	1.992	2.479	2.555
Y647S	M3 (sYtanlaaf)		1.443	2.196	1.905	3.151	2.091	3.065	1.546	2.200	3.799	2.285
N650K	M3 (sytaNlaaf)		1.977	3.402	1.997	2.669	2.363	2.553	2.305	2.467	2.213	1.666
E662K	S2 (drpeEr)		1.553	2.010	2.170	3.584	2.101	3.155	2.220	2.399	2.810	2.075
G815R	M4		1.627	2.357	1.835	4.124	2.532	2.992	1.670	2.448	2.937	2.373
G815V	M4		1.487	1.793	2.309	2.677	2.251	2.223	1.976	2.102	2.171	2.272
F817L	M4		2.066	2.820	2.279	2.634	2.211	2.628	2.355	2.428	2.014	1.601
G827R	M4		1.654	2.112	3.235	1.651	3.552	2.463	4.264	2.704	2.044	4.093
R844C	CTD		1.550	2.162	3.024	2.830	6.463	2.962	1.789	2.969	3.126	7.855
Polymicrogyria-associated *GRIN1* mutations												
L551P	S1-M1		1.030	0.370	2.085	3.249	3.529	3.174	2.471	2.273	2.600	2.998
S553L	S1-M1		1.578	0.452	2.584	3.266	1.91	3.107	2.199	2.157	2.255	1.37
Y647C	M3 (sYtanlaaf)		1.648	1.736	3.096	2.248	1.764	2.666	1.602	2.109	2.293	2.127
N650I	M3 (sytaNlaaf)		1.201	0.264	2.176	3.044	2.080	3.054	1.763	1.940	2.254	1.612
A653G	M3 (sytanlaAf)		1.276	0.471	3.052	3.338	2.591	2.799	2.338	2.266	2.584	1.057
R659W	S2 (dRpeer)		1.909	2.471	2.061	3.171	4.597	2.821	3.319	2.907	2.319	4.133
N674I	S2		1.693	2.087	2.290	3.605	5.246	2.766	1.850	2.791	4.596	6.199
D789N	S2		1.587	1.821	1.824	2.977	1.169	3.055	1.655	2.013	2.495	1.265
R794Q	S2		1.617	3.286	2.216	3.106	2.956	4.256	3.264	2.957	2.909	2.710
Mann–Whitney U test (*P*-value)			1.000	0.084	0.978	0.074	0.846	0.057	0.487	0.890	1.000	0.846

Root-mean-square deviation (Å)	less than 2	2 to 2.999	3 to 3.999	4 to 4.999	5 or more

Root-mean-square deviation (in Angstroms [Å]) for backbone atoms in specific domains, comparing mutant and wild-type GluN1. AT, amino terminal; S1-2, first and second ligand binding domains; M1-M4, transmembrane helices one to four; CBD, Ca^{2+}/calmodulin binding domain.

Supplementary Table 5. Molecular details and in silico predictions for the additional de novo mutations found in Patients 1-4.

Patient	Gene	GenBank accession ID	Location	cDNA/ Protein	Inherit-ance	dbSNP	ExAC	PhyloP	Grantham	SIFT	Mutation taster	PolyPhen-2	Scaled CADD	M-CAP
3	*CHRNB2* [a]	NM_000748.2	Chr1: g.154541944 G>A	c.71G>A p.(Trp24*)	*De novo*	-	-	-	-	-	-	-	Top 0.1% (35)	-
2	*CRIM1*	NM_016441.2	Chr2: g.36749247 G>A	c.2219G>A p.(Arg740Gln)	*De novo*	rs14240 1337	0.00 082%	0.53	43	Tolerated (1)	Polymorph ism (1)	Benign (0)	Top 10% (10.87)	Likely Benign (0.016)
2	*HIST1 H2BM*	NM_003521.2	Chr6: g.27783011 _27783012insT	c.190_191insT p.(Asn64Ilefs*9)	*De novo*	-	-	-	-	-	-	-	Top 1% (29.5)	-
1	*MEGF8* [b]	NM_001410.2	Chr16: g.42855697 G>A	c.2771G>A p.(Arg924Gln)	*De novo*	-	-	5.45	43	Deleterious (0)	Disease causing (1)	Probably damaging (0.995)	Top 0.1% (34)	-

a *CHRNB2* mutations are associated with Autosomal Dominant Nocturnal frontal lobe epilepsy type 3 (ADNFLE, OMIM 118507). This variant may have contributed to the patient's epilepsy phenotype. However, ADNFLE/*CHRNB2* mutations are not known to cause polymicrogyria.

b Mutations in *MEGF8* are associated with Carpenter syndrome (OMIM 604267). Carpenter syndrome (acrocephalopolysyndactyly type 2) is a rare autosomal recessive syndrome associated with craniofacial malformations, obesity and syndactyly. The patient did not have the clinical features of Carpenter syndrome and no second MEGF8 mutation was found in patient's exome data.
